# Biomarker-Based Prediction of Longitudinal Tau Positron Emission Tomography in Alzheimer Disease

**DOI:** 10.1001/jamaneurol.2021.4654

**Published:** 2021-12-20

**Authors:** Antoine Leuzy, Ruben Smith, Nicholas C. Cullen, Olof Strandberg, Jacob W. Vogel, Alexa Pichet Binette, Edilio Borroni, Shorena Janelidze, Tomas Ohlsson, Jonas Jögi, Rik Ossenkoppele, Sebastian Palmqvist, Niklas Mattsson-Carlgren, Gregory Klein, Erik Stomrud, Oskar Hansson

**Affiliations:** 1 Clinical Memory Research Unit, Department of Clinical Sciences, Lund University, Malmö, Sweden; 2 Department of Neurology, Skåne University Hospital, Lund, Sweden; 3 Penn/CHOP Lifespan Brain Institute, University of Pennsylvania, Philadelphia; 4 Department of Psychiatry, University of Pennsylvania, Philadelphia; 5 F. Hoffmann-La Roche Ltd, Basel, Switzerland; 6 Department of Radiation Physics, Skåne University Hospital, Lund, Sweden; 7 Department of Clinical Physiology and Nuclear Medicine, Skåne University Hospital, Lund, Sweden; 8 VU University Medical Center, Neuroscience Campus Amsterdam, Amsterdam, the Netherlands; 9 Memory Clinic, Skåne University Hospital, Lund, Sweden; 10 Wallenberg Centre for Molecular Medicine, Lund University, Lund, Sweden

## Abstract

**Importance:**

There is currently no consensus as to which biomarkers best predict longitudinal tau accumulation at different clinical stages of Alzheimer disease (AD).

**Objective:**

To describe longitudinal [^18^F]RO948 tau positron emission tomography (PET) findings across the clinical continuum of AD and determine which biomarker combinations showed the strongest associations with longitudinal tau PET and best optimized clinical trial enrichment.

**Design, Setting, and Participants:**

This longitudinal cohort study consecutively enrolled amyloid-β (Aβ)–negative cognitively unimpaired (CU) participants, Aβ-positive CU individuals, Aβ-positive individuals with mild cognitive impairment (MCI), and individuals with AD dementia between September 2017 and November 2020 from the Swedish BioFINDER-2 (discovery cohort) and BioFINDER-1 (validation cohort) studies.

**Exposures:**

Baseline plasma and cerebrospinal fluid Aβ42/Aβ40, tau phosphorylated at threonine-217 (p-tau217), p-tau181 and neurofilament light, magnetic resonance imaging, amyloid PET ([^18^F]flutemetamol), and tau PET ([^18^F]RO948 in the BioFINDER-2 study; [^18^F]flortaucipir in the BioFINDER-1 study).

**Main Outcomes and Measures:**

Baseline tau PET standardized uptake value ratio (SUVR) and annual percent change in tau PET SUVR across regions of interest derived using a data-driven approach combining clustering and event-based modeling. Regression models were used to examine associations between individual biomarkers and longitudinal tau PET and to identify which combinations best predicted longitudinal tau PET. These combinations were then entered in a power analysis to examine how their use as an enrichment strategy would affect sample size in a simulated clinical trial.

**Results:**

Of 343 participants, the mean (SD) age was 72.56 (7.24) years, and 157 (51.1%) were female. The clustering/event-based modeling–based approach identified 5 regions of interest (stages). In Aβ-positive CU individuals, the largest annual increase in tau PET SUVR was seen in stage I (entorhinal cortex, hippocampus, and amygdala; 4.04% [95% CI, 2.67%-5.32%]). In Aβ-positive individuals with MCI and with AD dementia, the greatest increases were seen in stages II (temporal cortical regions; 4.45% [95% CI, 3.41%-5.49%]) and IV (certain frontal regions; 5.22% [95% CI, 3.95%-6.49%]), respectively. In Aβ-negative CU individuals and those with MCI, modest change was seen in stage I (1.38% [95% CI, 0.78%-1.99%] and 1.80% [95% CI, 0.76%-2.84%], respectively). When looking at individual predictors and longitudinal tau PET in the stages that showed most change, plasma p-tau217 (*R^2^* = 0.27, *P* < .005), tau PET (stage I baseline SUVR; *R^2^* = 0.13, *P* < .05) and amyloid PET (*R^2^* = 0.10, *P* < .05) were significantly associated with longitudinal tau PET in stage I in Aβ-positive CU individuals. In Aβ-positive individuals with MCI, plasma p-tau217 (*R^2^* = 0.24, *P* < .005) and tau PET (stage II baseline SUVR; *R^2^* = 0.44, *P* < .001) were significantly associated with longitudinal tau PET in stage II. Findings were replicated in BioFINDER-1 using longitudinal [^18^F]flortaucipir. For the power analysis component, plasma p-tau217 with tau PET resulted in sample size reductions of 43% (95% CI, 34%-46%; *P* < .005) in Aβ-positive CU individuals and of 68% (95% CI, 61%-73%; *P* < .001) in Aβ-positive individuals with MCI.

**Conclusions and Relevance:**

In trials using tau PET as the outcome, plasma p-tau217 with tau PET may prove optimal for enrichment in preclinical and prodromal AD. However, plasma p-tau217 was most important in preclinical AD, while tau PET was more important in prodromal AD.

## Key Points

**Question:**

Which biomarkers best predict longitudinal tau accumulation at different clinical stages of Alzheimer disease?

**Findings:**

In this cohort study of 343 participants including amyloid-β–positive individuals who were cognitively unimpaired or had mild cognitive impairment, the largest annual increase in [^18^F]RO948 tau positron emission tomography (PET) was seen across the entorhinal cortex, hippocampus, and amygdala and in temporal cortical regions, respectively. In a power analysis, plasma phosphorylated tau217 with tau PET at baseline in stage I and II, respectively, resulted in sample size reductions.

**Meaning:**

In trials using tau PET as a main outcome, plasma phosphorylated tau217 with tau PET may prove optimal for enrichment in both preclinical and prodromal Alzheimer disease.

## Teaser

This cohort study describes longitudinal tau positron emission tomography findings using [^18^F]RO948 across the different clinical stages of Alzheimer disease.

## Introduction

Although differing hypotheses exist as to the molecular pathogenesis of Alzheimer disease (AD),^1,2,3^ a widely held view is that amyloid-β (Aβ) pathology potentiates the spread of tau through the neocortex with tau in turn associated with neurodegeneration, the proximal cause of clinical symptoms.^4,5^ Experimental and autopsy studies indeed provide support for this pathway, showing that tau tangles correlate strongly with neuronal and synaptic loss and cognitive decline.^6,7^ Further, using in vivo positron emission tomography (PET) and tau-specific tracers, cross-sectional studies have shown tracer retention to overlap closely with measures of brain hypometabolism and atrophy in AD-specific regions^8,9,10,11^ and to correlate with cognitive decline.^12,13,14,15^

Using [^18^F]flortaucipir, the most widely studied tau PET tracer to date, significant tracer retention has been observed in the basal ganglia, thalamus, and the choroid plexus, regions not shown to exhibit AD-like tau aggregates.^16,17,18^ As a result, novel tau PET tracers with reduced off-target binding in these regions have been developed,^19^ including [^18^F]PI-2620, [^18^F]GTP1, [^18^F]MK6240, and [^18^F]RO948. Longitudinal studies using [^18^F]flortaucipir^20,21,22,23,24^ and, more recently, [^18^F]MK6240^25^ have shown that tau accumulates in medial temporal areas in cognitively unimpaired (CU) individuals before spreading to cortical regions in individuals with cognitive impairment. In these studies, quantification of tau PET signal was performed using regions of interest (ROIs) developed using [^18^F]flortaucipir that approximate the neuropathology-based Braak staging system for tau neurofibrillary tangles.^15,26,27^ As yet, there are no published longitudinal findings using [^18^F]RO948 tau PET.

Increasingly, longitudinal tau PET is used as on outcome in AD clinical trials evaluating disease-modifying therapies. In anti-tau trials, tau PET should be used to determine target engagement, which is an important step already during the early phases of drug development to avoid large-scale phase 3 trials with drugs unlikely to result in a positive clinical outcome.^4^

Further, longitudinal tau PET is nowadays often used to study the effects of drugs directed against more upstream events in AD, such as Aβ, including whether such therapies can slow the accumulation and spread of tau, which could indicate a positive clinical effect beyond just target engagement.^28^ However, to use longitudinal tau PET effectively as an outcome in clinical trials, enrichment for individuals likely to show tau accumulation and spread during the trial is key. This increases the statistical power to detect treatment effects. Although sample enrichment can be based on baseline biomarker profiles, there are no studies yet that have systematically addressed which imaging and fluid biomarkers are most strongly associated with longitudinal tau PET at different clinical stages of AD.

Here, we aimed to describe longitudinal tau PET findings using [^18^F]RO948 across the different clinical stages of AD. Next, we determined the association between baseline fluid and imaging AD biomarkers for Aβ, tau, and neurodegeneration^29,30^ and longitudinal tau PET. Findings from this step were then entered in a power analysis to determine how biomarker driven enrichment would affect sample size in a simulated clinical trial using a 30% reduction in annual change in tau PET as outcome. A data set with longitudinal [^18^F]flortaucipir (BioFINDER-1) was also included as an independent validation cohort.

## Methods

### Participants

We included participants from the prospective and longitudinal Swedish BioFINDER-2 study (NCT03174938), including CU individuals and patients with mild cognitive impairment (MCI) and AD dementia. Participants were enrolled between September 2017 and November 2020. Inclusion/exclusion criteria have been described elsewhere (eMethods 1 in the Supplement).^31,32^ CU individuals were 60 years or older and did not have MCI or dementia.^31,32^ Exclusion criteria included presence of objective cognitive impairment, severe somatic disease, and current alcohol/substance misuse. Patients with MCI fulfilled the *Diagnostic and Statistical Manual of Mental Disorders* (Fifth Edition) criteria for mild neurocognitive disorder while patients with AD dementia fulfilled the *Diagnostic and Statistical Manual of Mental Disorders* (Fifth Edition) criteria for major cognitive impairment due to AD.^33^ Aβ status was defined using amyloid PET and a previous established threshold (Centiloid ≥19) beyond which amyloid PET has been shown to reliably increase.^33^ Sensitivity analyses using all CU individuals and individuals with MCI regardless of Aβ status are reported in eTables 1-3 in the Supplement. All participants gave written informed consent. Ethical approval was given by the Regional Ethical Committee in Lund, Sweden. Approval for PET imaging was obtained from the Swedish Medicines and Products Agency and the local Radiation Safety Committee at Skåne University Hospital in Sweden.

### Plasma and Cerebrospinal Fluid Biomarkers

Plasma Aβ42/Aβ40 was measured using a mass spectrometry-based assay (Araclon Biotech).^34^ Phosphorylated tau (p-tau)217 (cerebrospinal fluid [CSF] and plasma) was measured on a Meso Scale Discovery platform, using an assay developed by Eli Lilly.^35,36^ Plasma neurofilament light was analyzed using a Simoa-based assay.^37^ CSF Aβ42/40 and neurofilament light were measured using the Elecsys platform (Roche).^38^ Main analyses were performed using plasma biomarkers with findings using CSF-based biomarkers reported in eTables 3 to 5 in the Supplement. Sensitivity analyses were also performed using p-tau181, measured at Eli Lilly using the Meso Scale Discovery platform (CSF)^35^ and an in-house Simoa-based immunoassay at the Clinical Neurochemistry Laboratory in Gothenburg (plasma).^39^

### Image Acquisition and Processing

T1-weighted magnetic resonance images were acquired on a 3-T MAGNETOM Prisma scanner (Siemens Healthineers) for PET image coregistration and template normalization. FreeSurfer version 6.0 was used to extract hippocampal volume and cortical thickness within a meta-ROI encompassing temporal regions with known susceptibility in AD (AD cortex: mean thickness in bilateral entorhinal, inferior temporal, middle temporal, and fusiform cortices).^40^

[^18^F]RO948 PET was performed 70 to 90 minutes postinjection (list-mode acquisition), as described previously.^31^ Standardized uptake value ratio (SUVR) images were created using the inferior cerebellar cortex as reference region. To extract mean regional SUVR values, FreeSurfer-based parcellation of the T1-weighted magnetic resonance imaging scan was applied to the [^18^F]RO948 data transformed to participants’ native T1 space. As some off-target binding in the choroid plexus has been described for [^18^F]RO948,^41^ analyses were performed using partial-volume–corrected (Geometric Transfer Matrix) data.^42^ For [^18^F]flutemetamol PET, data were acquired 90 to 110 minutes postinjection, as detailed elsewhere.^43^ After coregistration to their corresponding magnetic resonance imaging, [^18^F]flutemetamol images were spatially normalized to template space. SUVR values were then obtained using a neocortical meta-ROI and the whole cerebellum as reference and converted to Centiloids using the Computational Analysis of PET from AIBL (CapAIBL) pipeline.^44^

### Tau PET ROI Definition

As the properties of [^18^F]RO948 differ from those of [^18^F]flortaucipir,^41^ we derived [^18^F]RO948-specific ROIs using a data-driven approach combining clustering and event-based modeling (EBM),^45,46^ an approach that allows for the ordering of biomarker changes and that has previously been applied to [^18^F]flortaucipir.^47^ Primary analyses were performed using clustering/EBM–derived ROIs (eFigure in the Supplement). For the clustering component, where the aim was to determine stable patterns of [^18^F]RO948 signal covariance, a 2-component Gaussian mixture model was first applied to [^18^F]RO948 SUVR data for each of the bilateral FreeSurfer ROIs using all available baseline scans in BioFINDER-2 (464 CU, 196 MCI, 150 AD dementia, and 216 non-AD). The predicted probability of the abnormal component was calculated for each sample, effectively converting regional SUVR values to regional tau-positive probabilities (ie, the probability that a participant’s SUVR value falls within the rightmost portion of the Gaussian distribution,^47^ representing abnormal [^18^F]RO948 signal). An unsupervised consensus clustering algorithm (bootstrap analysis of stable clusters) was then applied to cluster the data,^48,49^ with a 5-cluster solution adopted based on silhouette scores. Next, we used EBM to derive the order in which each of the 5 clusters become abnormal. In EBM, an event represents the transition from a normal to an abnormal state, with the EBM determining the event sequence that maximizes the data likelihood (ie, the most likely ordering of the events). Regional tau-positive probabilities were averaged within cluster-derived ROIs and submitted to EBM, using 10 000 Monte-Carlo simulations to derivate uncertainty in event ordering.

A sensitivity analysis was performed using [^18^F]flortaucipir–derived Braak ROIs.^26^ Further, as the transentorhinal cortex (Brodmann area 35) is considered the earliest site of tau pathology on the basis of postmortem data,^50,51,52^ an additional analysis was performed using baseline SUVR in this region^53^ as the predictor of EBM-based change in Aβ-positive CU individuals.

### Independent Validation Cohort

Eighty-five participants with available [^18^F]flutemetamol PET, magnetic resonance imaging and fluid biomarkers, as well as longitudinal [^18^F]flortaucipir PET, were included from the Swedish BioFINDER-1 study as a validation cohort. Inclusion and exclusion criteria are described elsewhere^54^ (eMethods 2 in the Supplement). As p-tau217 was not available in these participants, p-tau181 measured with an immunoassay developed by Lilly Research Laboratories^35^ was used. CSF Aβ42/40 was measured using the Euroimmun assay,^55^ while plasma Aβ42/40 and neurofilament light (CSF and plasma) were measured using Roche Elecsys platform.

### Statistical Analyses

All analyses were performed in R version 4.0.4 (R Foundation). Two-sided *P *<* *.05 indicated statistical significance. First, annual change in tau PET SUVR was determined between baseline and follow-up within EBM-based ROIs. This was calculated as the difference between follow-up and baseline, divided by baseline uptake and multiplied by the time interval between scans in years: ([follow-up SUVR – baseline SUVR] / baseline SUVR) × 100 / ∆time.

Having calculated longitudinal change in tau PET, regression models were used to examine whether individual biomarkers (plasma Aβ42/40, amyloid PET, plasma p-tau217, baseline tau PET in the outcome EBM stage, plasma neurofilament light, hippocampal volume, and AD cortex) were significantly associated with change in [^18^F]RO948 SUVR. This analysis was performed groupwise (ie, Aβ-positive CU and Aβ-positive MCI) using annual percent change in the EBM ROI that showed the highest change in tau PET SUVR for a given group as the response variable. Models were compared using coefficient of determination (*R^2^*) and Akaike information criterion (AIC; lower indicates better) values relative to a demographics only model (age and sex).

In addition to testing individual predictors, we used a backward stepwise model selection approach to identify the best combination of biomarkers. Here, the best-fitting model (ie, best combination) was that which included the fewest predictors among the models within 2 points of the lowest AIC value; this procedure is well established for selecting the most parsimonious model based on AIC values.^56,57^ Lastly, the best biomarker combination was used in a regression-based power analysis to calculate the reduction in sample size needed to observe a 30% reduction in the annual percentage change in tau PET SUVR. Model fit was assessed using change in AIC relative to inclusion of all participants (ie, no enrichment), assuming selection of individuals with biomarker *z* scores of 2 or higher (*z* score transformed using Aβ-negative CU individuals; eTable 6 in the Supplement).

## Results

### Participant Characteristics and Change in Tau PET

**Figure 1.**
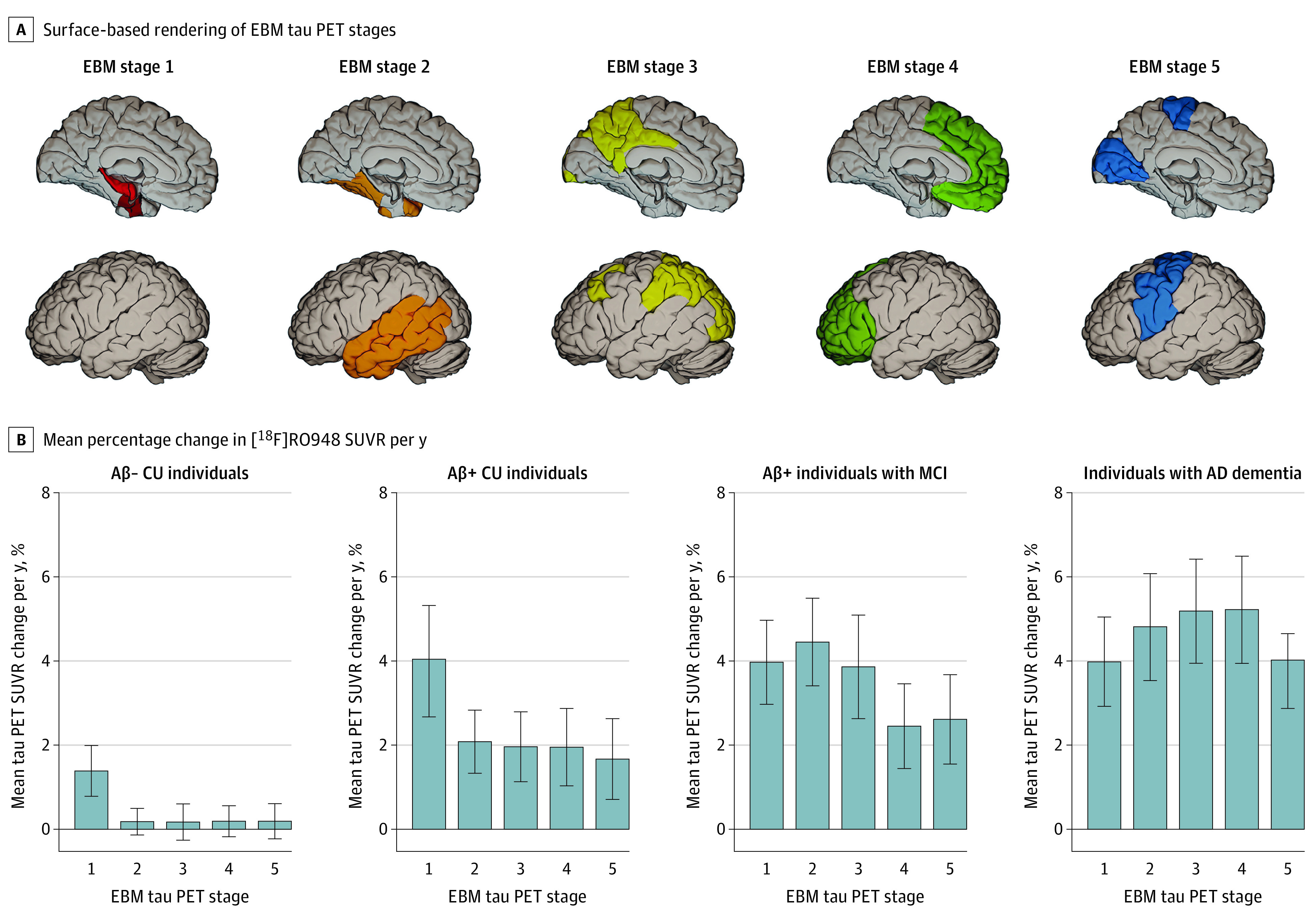
EBM-Based Regions of Interest and Annual Percent Change in Tau PET Standardized Uptake Value by EBM Stage A, Surface-based rendering of event-based modeling (EBM) tau positron emission tomography (PET) stages (I through V, left to right). B, Mean percent change in [^18^F]RO948 standardized uptake value ratio per year along with 95% CIs. Aβ indicates amyloid-β; CU, cognitively unimpaired; MCI, mild cognitive impairment; p-tau, phosphorylated tau

A total of 343 participants with longitudinal tau PET were included (137 Aβ-negative CU individuals [40.0%], 49 Aβ-positive CU individuals [14.3%], 36 Aβ-negative individuals with MCI [10.5%], 58 Aβ-positive individuals with MCI [16.9%], and 63 individuals with AD dementia [18.4%]). Across all participants, the mean (SD) age was 72.56 (7.24) years, and 157 (51.1%) were female. Participant characteristics are summarized in Table 1 and eTable 1 in the Supplement (for Aβ-negative individuals with MCI and for all CU individuals and those with MCI regardless of Aβ status). The 5 EBM-based clusters (stages) are shown in Figure 1A. In Aβ-positive CU individuals, the largest annual increase in [^18^F]RO948 SUVR was seen in stage I (entorhinal cortex, hippocampus, and amygdala: 4.04% [95% CI, 2.76%-5.32%]); in Aβ-positive individuals with MCI, the greatest change was seen in stage II (temporal cortical regions: 4.45% [95% CI, 3.41%-5.49%]), while in individuals with AD dementia, the greatest change was seen in stage IV (certain frontal regions: 5.22% [95% CI, 3.95%-6.49%]) (Figure 1B). Modest change was seen in Aβ-negative CU and MCI groups using stage I (1.38% [95% CI, 0.80%-1.96%] and 1.80% [95% CI, 0.76%-2.84%], respectively). Using 1-sample *t* tests, changes across EBM-based ROIs were significant except for stages II through V in the Aβ-negative CU and MCI groups. These findings, along with minimum and maximum percentage change values are summarized in eTable 7 in the Supplement. Findings using Braak ROIs (eTable 8 in the Supplement) were similar to those using EBM-based ROIs. Findings in all CU individuals and individuals with MCI regardless of Aβ status are reported in eTable 8 in the Supplement.

**Table 1.** Participant Characteristics Including A/T/N Biomarkers and Longitudinal Tau PET

Characteristic	Mean (SD)			
	Aβ- CU individuals	Aβ+ CU individuals	Aβ+ individuals with MCI	Individuals with AD dementia
No.	137	49	58	63
Age, y	72.57 (7.33)	72.83 (7.52)	71.79 (7.97)	73.06 (6.90)
Female, No. (%)	64 (47)	26 (53)	32 (55)	35 (56)
Male, No. (%)	73 (53)	23 (47)	26 (45)	28 (44)
Education, y	12.78 (3.25)	12.15 (4.07)	13.15 (5.24)	11.92 (4.65)
MMSE score	28.98 (1.21)	28.67 (1.30)	26.69 (1.91)	19.74 (4.23)
APOE ε4 carrier, No. (%)	52 (37)	41 (84)	37 (63)	40 (63)
Tau PET, scan interval, mean (SD) [95% CI], y	1.79 (0.19) [1.12 to 2.07]	1.78 (0.19) [0.85 to 1.95]	1.72 (0.24) [0.85 to 2.11]	1.54 (0.32) [0.73 to 2.06]
Baseline tau PET, SUVR				
EBM stage				
I	0.97 (0.13)	1.17 (0.21)	1.38 (0.32)	1.71 (0.36)
II	1.27 (0.11)	1.42 (0.39)	1.61 (0.45)	2.62 (0.94)
III	1.26 (0.12)	1.35 (0.36)	1.55 (0.54)	2.37 (0.91)
IV	1.13 (0.11)	1.16 (0.17)	1.20 (0.18)	1.69 (0.86)
V	1.18 (0.11)	1.19 (0.12)	1.20 (0.14)	1.52 (0.43)
Annual change tau PET SUVR, mean (95% CI), %				
EBM stage				
I	1.38 (0.80 to 1.96)	4.04 (2.76 to 5.32)	3.97 (2.97 to 4.97)	3.98 (2.92 to 5.04)
II	0.18 (−0.14 to 0.49)	2.31 (1.33 to 2.83)	4.45 (3.41 to 5.49)	4.81 (3.54 to 6.08)
III	0.17 (−0.26 to 0.60)	2.21 (1.38 to 3.04)	3.87 (2.63 to 5.09)	5.18 (3.94 to 6.42)
IV	0.19 (−0.18 to 0.56)	2.17 (1.25 to 3.09)	2.45 (1.44 to 3.46)	5.22 (3.95 to 6.49)
V	0.13 (−0.19 to 0.65)	1.67 (0.71 to 2.63)	2.61 (1.55 to 3.67)	4.02 (2.87 to 5.17)
A/T/N variables				
Aβ42/40				
Plasma	0.22 (0.03)	0.20 (0.03)	0.18 (0.04)	0.19 (0.04)
CSF	0.11 (0.02)	0.05 (0.01)	0.02 (0.03)	0.04 (0.01)
Amyloid PET, Centiloids	−8.11 (6.33)	50.72 (21.27)	60.88 (25.44)	NA^a^
P-tau217, pg/mL				
Plasma	1.29 (2.67)	3.22 (2.39)	3.81 (2.14)	8.01 (4.38)
CSF	42.69 (27.21)	245.40 (93.30)	258.63 (133.92)	599.49 (320.36)
NfL, pg/mL				
Plasma	12.40 (7.38)	17.65 (5.71)	18.91 (9.80)	28.47 (36.10)
CSF	108.50 (51.93)	201.70 (143.16)	197.90 (94.37)	315.04 (284.61)
Hippocampal volume, mm^3^	3874.64 (437.76)	3636.59 (513.74)	3268.75 (520.90)	2799.20 (421.44)
AD cortex thickness, mm	2.76 (0.12)	2.62 (0.16)	2.52 (0.15)	2.38 (0.20)

Abbreviations: Aβ, amyloid-β; Aβ42/40, ratio of amyloid-β42 to amyloid-β40; AD, Alzheimer disease; AD cortex, cortical thickness in a temporal meta region of interest encompassing temporal regions with known susceptibility in Alzheimer disease; APOE, apolipoprotein E; A/T/N, amyloid/tau/neurodegeneration; EBM, event-based modeling; CSF, cerebrospinal fluid; CU, cognitively unimpaired; MCI, mild cognitive impairment; MMSE, Mini-Mental State Examination; NA, not applicable; NfL, neurofilament light; PET, positron emission tomography; p-tau217, tau phosphorylated at threonine 217; SUVR, standardized uptake value ratio.

^a^ In BioFINDER-2, amyloid PET is by design performed only in individuals without cognitive impairment and those with MCI.

### Modeling Change in Tau PET Using Individual Predictors

Next, we tested whether individual biomarkers were significantly associated with annual change in [^18^F]RO948 tau PET SUVR (Table 2). These analyses were performed in Aβ-positive CU individuals and those with MCI as these populations are of primary interest for AD clinical trials. In Aβ-positive CU individuals, plasma p-tau217 (*R^2^* = 0.27; *P* < .005; ΔAIC = −10), tau PET (baseline SUVR in stage I, *R^2^* = 0.13; *P* < .05; ΔAIC = −3) and amyloid PET (*R^2^* = 0.10; *P* < .05; ΔAIC = −2) showed significant associations with annual change in [^18^F]RO948 SUVR in stage I. In Aβ-positive individuals with MCI, plasma p-tau217 (*R^2^* = 0.24; *P* < .001; ΔAIC = −10) and tau PET (baseline SUVR in stage II, *R^2^* = 0.33; *P* < .001; ΔAIC = −25) were significantly associated with annual changes in [^18^F]RO948 SUVR in stage II. Similar findings were seen when using CSF instead of plasma-based biomarkers (eTable 4 in the Supplement) and when using p-tau181 instead of p-tau217 (eTables 9 and 10 in the Supplement). Results were consistent with EBM-based findings when using Braak ROIs (eTable 11 in the Supplement) and when using baseline SUVR in Brodmann area 35 instead of the stage I ROI in Aβ-positive CU individuals (eTable 12 in the Supplement). Findings in all CU individuals and those with MCI regardless of Aβ status are reported in eTables 2 and 3 in the Supplement. Findings for AD dementia, where plasma p-tau217, CSF p-tau217, and tau PET (baseline SUVR in stage IV) were significantly associated with annual changes in [^18^F]RO948 SUVR in stage IV are summarized in eTables 5 and 13 in the Supplement.

**Table 2.** Associations Between Individual Variables and Longitudinal Tau PET in CU Individuals and Individuals With MCI With Evidence of Aβ Pathology

Group	Variable	Coefficient	Adjusted *R^2 ^*(95% CI)	*P* value	∆AIC
Aβ+ CU individuals^a^^,^^b^	Plasma Aβ42/40	−0.03	0.00 (−0.02 to 0.02)	.86	2
	Amyloid PET	0.32	0.10 (−0.07 to 0.27)	.047	−2
	Plasma p-tau217	0.53	0.27 (0.04 to 0.49)	<.005	−10
	Tau PET	0.36	0.13 (−0.06 to 0.31)	.03	−3
	Plasma NFL	0.10	0.01 (−0.05 to 0.07)	.57	2
	Hippocampal volume	0.06	0.00 (−0.03 to 0.04)	.72	2
	AD cortex	−0.07	0.01 (−0.04 to 0.05)	.67	2
Aβ+ individuals with MCI^c^^,^^b^	Plasma Aβ42/40	−0.24	0.05 (−0.06 to 0.17)	.12	−1
	Amyloid PET	0.16	0.02 (−0.06 to 0.11)	.30	1
	Plasma p-tau217	0.49	0.24 (0.04 to 0.44)	<.001	−10
	Tau PET	0.67	0.44 (0.16 to 0.57)	<.001	−25
	Plasma NFL	−0.07	0.00 (−0.03 to 0.04)	.67	2
	Hippocampal volume	−0.18	0.03 (−0.06 to 0.13)	.23	0
	AD cortex	−0.16	0.02 (−0.06 to 0.11)	.31	1

Abbreviations: ∆AIC, change in Akaike information criterion relative to a demographics only model (age and sex); Aβ, amyloid-β; Aβ42/40, ratio of amyloid-β42 to amyloid-β40; AD cortex, cortical thickness in a temporal meta region of interest encompassing temporal regions with known susceptibility in Alzheimer disease; CU, cognitively unimpaired; MCI, mild cognitive impairment; NfL, neurofilament light; PET, positron emission tomography; p-tau217, tau phosphorylated at threonine 217.

^a^ Tau PET refers to [^18^F]RO948 standardized uptake value ratio at baseline in the event-based modeling stage I region of interest.

^b^ Amyloid PET refers to the standardized uptake value ratio at baseline in a neocortical meta region of interest, expressed in Centiloids.

^c^ Tau PET refers to [^18^F]RO948 standardized uptake value ratio at baseline in the event-based modeling stage II region of interest.

### Power Analysis for Theoretical Clinical Trial

**Figure 2.**
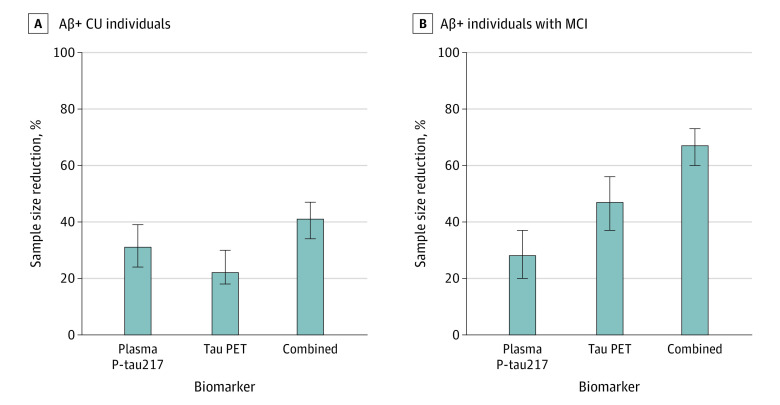
Power Enrichment Analysis of Plasma Biomarkers in a Theoretical Clinical Trial Using Tau PET as End Point Mean percent sample size reductions along with 95% CIs are shown for individual biomarkers and combined in Aβ-positive CU individuals (A) and in Aβ-positive individuals with MCI (B). Aβ indicates amyloid-β; AD, Alzheimer disease; CU, cognitively unimpaired; EBM, event-based modeling; MCI, mild cognitive impairment; PET, positron emission tomography; SUVR, standardized uptake value..

Lastly, we tested which biomarker combinations were most strongly associated with annual increases in [^18^F]RO948 SUVR and their association with sample size when used for population enrichment of participants that will exhibit a more positive slope in tau PET over time. Basing selection on the most parsimonious model (ie, including the least number of biomarkers and having an AIC within 2 points of the lowest AIC value), a model combining plasma p-tau217 and baseline tau PET proved best in Aβ-positive CU individuals (*R^2^* = 0.31, AIC = 100) and in Aβ-positive individuals with MCI (*R^2^* = 0.46, AIC = 111) (Table 3). Applying these models for population enrichment in a clinical trial context resulted in sample size reductions of 43% (95% CI, 34%-47%; *P* < .005; ∆AIC = −2) in Aβ-positive CU individuals and 68% (95% CI, 60%-73%; *P* < .001; AIC = −10) in Aβ-positive individuals with MCI (Figure 2). When examining the importance of individual predictors, sample size reductions were larger using plasma p-tau217 (31% [95% CI, 24%-39%]; *P* < .005; ∆AIC = −5) compared with tau PET (22% [95% CI, 16%-29%]; *P* < .05; ∆AIC = −2) in Aβ-positive CU individuals, and larger using tau PET (47% [95% CI, 37%-56%]; *P* < .001; ∆AIC = −10) compared with plasma p-tau217 (28% [95% CI, 30%-37%]; *P* < .05; ∆AIC = −2) in Aβ-positive individuals with MCI. Results using all CU and MCI independent of Aβ status are reported in eTables 14 and 15 in the Supplement. Results for AD dementia are summarized in eTables 16 and 17 in the Supplement.

**Table 3.** Summary of Model Selection Approach for Variables Using Longitudinal Tau PET as Outcome

Group	Model	Removed from model	*R^2^*	AIC
Aβ+ CU individuals^a^	Plasma Aβ42/40, amyloid PET, plasma p-tau217, tau PET, plasma NFL, hippocampal volume, AD cortex	NA	0.33	108
	Plasma Aβ42/40, amyloid PET, plasma p-tau217, tau PET, hippocampal volume, AD cortex	Plasma NFL	0.33	106
	Plasma Aβ42/40, amyloid PET, plasma p-tau217, tau PET, AD cortex	Hippocampal volume	0.33	104
	Plasma Aβ42/40, amyloid PET, plasma p-tau217, tau PET	AD cortex	0.33	102
	Plasma Aβ42/40, tau PET, plasma p-tau217	Amyloid PET	0.32	101
	Tau PET, plasma p-tau217	Plasma Aβ42/40	0.31	100
	Plasma p-tau217	Tau PET	0.29	103
Aβ+ individuals with MCI^b^	Plasma Aβ42/40, amyloid PET, plasma p-tau217, tau PET, plasma NFL, hippocampal volume, AD cortex	NA	0.48	120
	Plasma Aβ42/40, amyloid PET, plasma p-tau217, tau PET, plasma NFL, hippocampal volume	AD cortex	0.48	118
	Plasma Aβ42/40, amyloid PET, plasma p-tau217, tau PET, plasma NFL	Hippocampal volume	0.48	116
	Plasma Aβ42/40, amyloid PET, plasma p-tau217, tau PET	Plasma NFL	0.48	115
	Plasma Aβ42/40, plasma P-tau217, tau PET	Amyloid PET	0.46	113
	Plasma p-tau217, tau PET	Plasma Aβ42/40	0.46	111
	Tau PET	Plasma p-tau217	0.45	114

Abbreviations: Aβ, amyloid-β; AD cortex, cortical thickness in a temporal meta region of interest encompassing temporal regions with known susceptibility in Alzheimer disease; AIC, Akaike information criterion; Aβ42/40, ratio of amyloid-β42 to amyloid-β40; CU, cognitively unimpaired; MCI, mild cognitive impairment; NA, not applicable; NfL, neurofilament light; PET, positron emission tomography; p-tau217, tau phosphorylated at threonine 217.

^a^ Tau PET refers to [^18^F]RO948 standardized uptake value ratio at baseline in the event-based modeling stage I region of interest.

^b^ Tau PET refers to [^18^F]RO948 standardized uptake value ratio at baseline in the event-based modeling stage II region of interest.

### Independent Validation Cohort

Among 85 study participants (12 Aβ-negative CU individuals, 27 Aβ-positive CU individuals, 10 Aβ-negative individuals with MCI, 16 Aβ-positive individuals with MCI, and 20 individuals with AD dementia) in the independent validation cohort, the mean (SD) age was 77.18 (7.74) years and 41 (48.32%) were female. Demographic and biomarker characteristics are shown in eTable 18 in the Supplement.

Similar to findings in BioFINDER-2, the largest annual increase in [^18^F]flortaucipir SUVR was seen in stage I (3.44% [95% CI, 2.96%-4.84%]) in Aβ-positive CU individuals, with Aβ-positive individuals with MCI and AD dementia showing the largest increases in stages II (4.35% [95% CI, 3.92%-4.78%]) and IV (4.21% [95% CI, 3.82%-4.60%]), respectively (eTable 18 in the Supplement). In Aβ-negative CU individuals, modest change was seen in stage I (1.39% [95% CI, 1.07%-1.71%]). When examining the associations between individual biomarkers and annual change in tau PET SUVR, plasma p-tau181 (*R^2^* = 0.21; *P* < .05; ΔAIC = −5), tau PET (*R^2^* = 0.14; *P* < .05; ΔAIC = −2), and amyloid PET (*R^2^* = 0.13; *P* < .05; ΔAIC = −2) showed significant associations with annual change in [^18^F]flortaucipir SUVR in stage I (eTable 19 in the Supplement). In Aβ-positive individuals with MCI, plasma p-tau181 (*R^2^* = 0.16; *P* < .05; ΔAIC = −2) and tau PET (*R^2^* = 0.44; *P* < .005; ΔAIC = −6) were significantly associated with annual change in [^18^F]flortaucipir SUVR in stage II (eTable 19 in the Supplement). Similar results were obtained when using CSF p-tau181 (eTable 20 in the Supplement).

## Discussion

Using a novel data-driven approach (EBM), we identified target ROIs that were broadly consistent with widely used Braak-like stages.^15,26,27^ Overall, these results support earlier work using [^18^F]flortaucipir^14,22,58,59,60^—and, more recently, [^18^F]MK6240^25^—showing that the accumulation of pathological tau was seen mainly in the medial temporal lobe early in the disease process (ie, Aβ-positive CU individuals) and primarily in neocortical areas in Aβ-positive individuals with cognitive impairment. The EBM-based stage I ROI encompassed the entorhinal cortex, the hippocampus, and the amygdala. In head-to-head work comparing [^18^F]RO948 and [^18^F]flortaucipir,^41^ [^18^F]RO948 showed higher signal in the entorhinal cortex and less off-target binding in the choroid plexus, making the hippocampus a region more suitable for use with [^18^F]RO948 compared with [^18^F]flortaucipir. The inclusion of the amygdala in the stage I ROI is consistent with previous work using [^18^F]flortaucipir^61^—and, more recently, [^18^F]RO948^62^ and [^18^F]MK6240^63^—identifying it as a site of early tau accumulation. The levels of annual change in tau PET SUVR in the stage I ROI were similar across Aβ-positive groups. Consistent with the Braak model of tau pathology in AD,^51^ this suggests that tau continues to accumulate in areas involved early on even after spreading into later regions.^24,64,65^ As elevated rates of tau accumulation in the neocortex were only seen in the presence of abnormal Aβ, our findings are consistent with earlier cross-sectional findings showing that abnormal [^18^F]RO948 SUVR levels were only seen in the context of elevated Aβ^31^ and support the idea that amyloidosis is an upstream driver of tau accumulation.^5,24,25,58^ However, findings in Aβ-negative CU, where a modest increase in medial temporal signal (stage I) was seen, suggest that [^18^F]RO948 may be able to detect primary age-related tauopathy.^2,66^

In Aβ-positive CU individuals, plasma p-tau217 was the predictor most strongly associated with longitudinal change in tau PET, followed by baseline tau PET and amyloid PET. Recent cross-sectional work examining plasma p-tau217 in CU persons showed that levels are elevated in individuals with signs of Aβ pathology,^32^ with high levels at baseline associated with larger increases in tau PET signal in the medial temporal lobe,^67^ and largely mediated the association between amyloid and tau PET.^67,68^ Similar findings have also been reported for CSF p-tau217.^69^ On the basis of these findings, it has been proposed that Aβ pathology is associated with an increase in the release and phosphorylation of tau. This disruption in the metabolism of soluble tau is reflected in elevated CSF and plasma p-tau and might be associated with the subsequent accumulation of tau aggregates. This scenario, whereby elevated Aβ levels result in increased plasma p-tau and subsequent accumulation of tau aggregates over time would account for the significant associations seen between longitudinal tau PET, and baseline plasma p-tau217, amyloid PET, and tau PET. In the combination analysis, plasma p-tau217 and tau PET proved the best combination, but baseline [^18^F]RO948 SUVR in stage I only showed a slightly stronger association to longitudinal tau PET than amyloid PET. This finding is in line with recent work that also showed a very similar degree of association between amyloid PET and tau PET with tau accumulation rates in CU individuals.^70^ In prodromal AD (ie, Aβ-positive individuals with MCI), baseline tau PET followed by plasma p-tau217 were the predictors most strongly associated with longitudinal tau PET. Our findings for tau PET align with previous findings showing a strong association between neocortical tau PET increase and baseline tau burden in Aβ-positive individuals with MCI.^22^ Once outside the medial temporal lobe, the baseline burden of tau aggregates appears to be the strongest predictor of future increases. The significant association of plasma p-tau217 with longitudinal tau PET at this stage, coupled with continued associations with tau PET in AD dementia, suggest that the disruption of soluble tau, as reflected by elevated plasma p-tau217 levels, may roughly parallel tau accumulation across the symptomatic course of AD.^69,71^ Although we were not able to assess other p-tau variants such as p-tau231,^72^ we observed very similar results between p-tau181 and p-tau217, consistent with recent work showing that both variants perform similarly when differentiating individuals based on tau PET status.^73^

Having established which biomarkers best predicted tau accumulation in Aβ-positive CU individuals and those with MCI, we assessed their association as enrichment approaches in the context of inclusion in clinical trials where longitudinal tau PET is used as a main outcome to detect either target engagement or clinical efficacy. Although our findings suggest that plasma p-tau217 and tau PET could be used as straightforward approaches to identify CU individuals and those with MCI, respectively, who are more likely to have high rates of tau accumulation, the greater sample size reductions seen when combining plasma p-tau217 with tau PET suggest that this approach may be favorable. This may prove cost-effective in trials involving preclinical and prodromal AD given that a baseline tau PET scan would anyway be required if using longitudinal tau PET as an end point and that plasma p-tau217 is comparatively easy and inexpensive to measure. While biomarker-based enrichment in clinical trials may increase statistical power, it also increases the number of screening failures. The cost associated with excluding individuals based on their baseline biomarker findings (ie, screening failures) may be justified by the risk of including participants who do not show longitudinal increase in tau PET. Future cost-focused studies are required to address these and related trade-offs.

### Strengths and Limitations

Strengths of this study include that we directly compared the predictive ability of many relevant imaging and fluid-based AD biomarkers, which has not been done before. Further, tau accumulation was measured longitudinally and compared using data (EBM) and postmortem-based (Braak) approaches. In addition to our study covering the clinical continuum of AD, our follow-up interval was comparatively long and similar across group, with Aβ status defined using Centiloids and a well-determined cut point. Importantly, we replicated our main findings using longitudinal [^18^F]flortaucipir PET in an independent cohort (BioFINDER-1). Limitations include the modest number of participants in the AD dementia and Aβ-positive groups and the fact that amyloid PET was not available for participants with AD dementia. An additional limitation specific to our power analysis was that we could not estimate within-participant measurement error because participants only had 2 tau PET scans. Although our focus was on establishing which biomarkers best predicted longitudinal change in the pre- and early symptomatic stages of AD, there remains interest in the testing of anti-tau compounds in patients with mild dementia due to AD.^74^ In addition, we cannot exclude that Aβ, tau, and neurodegeneration biomarkers or model types, including those incorporating nonlinear relationships and interactions between biomarkers, other than those investigated here may have resulted in better model fits. Lastly, future studies must address the importance of cutoff selection and relevant variables such as age. Younger participants, for instance, may be excluded owing to low or borderline levels of p-tau217 and baseline tau PET yet be on the verge of showing significant increases in tau levels.

## Conclusions

Although further work addressing optimal biomarker combinations is required, our results indicate that plasma p-tau217 and tau PET may significantly reduce sample sizes in preclinical and prodromal AD clinical trials using tau PET as one of the main outcomes. Although their combination provided the greatest sample size reduction, plasma p-tau217 was more important in preclinical AD, while tau PET was more important in prodromal AD.

## Notes

Supplement. **eMethods 1.** Inclusion and exclusion criteria for the Swedish BioFINDER-2 study. **eMethods 2.** Inclusion and exclusion criteria for the Swedish BioFINDER-1 study. **eFigure.** Overview of the steps involved in the clustering and EBM derived ROIs. **eTable 1.** Participant characteristics including A/T/N biomarkers and longitudinal. tau PET in Aβ-negative MCI and CU and MCI regardless of Aβ status. **eTable 2.** Relationship between individual biomarkers and tau PET using EBM ROIs and plasma biomarkers in all CU and MCI independent of Aβ status. **eTable 3.** Relationship between individual biomarkers and tau PET using EBM ROIs and CSF biomarkers in all CU and MCI independent of Aβ status. **eTable 4.** Relationship between individual biomarkers and tau PET using CSF biomarkers. **eTable 5.** Relationship between individual biomarkers and tau PET using EBM ROIs and CSF biomarkers in AD dementia. **eTable 6.** Characteristics of Aβ-negative CU individuals used for z-score transformation. **eTable 7.** Range of annual percent change values and results of one-sample t-tests across groups and EBM ROIs. **eTable 8.** Mean annual percent change in tau PET SUVR using Braak ROIs. **eTable 9.** Relationship between individual biomarkers and tau PET using plasma p-tau181. **eTable 10.** Relationship between individual biomarkers and tau PET using CSF p-tau181. **eTable 11.** Relationship between individual biomarkers and tau PET using Braak I ROI for Aβ-positive CU and Braak III ROI in Aβ-positive MCI. **eTable 12.** Relationship between individual biomarkers and tau PET in Aβ-positive CU using transentorhinal SUVR at baseline instead of EBM I baseline SUVR. **eTable 13.** Relationship between individual biomarkers and tau PET using EBM ROIs and plasma biomarkers in AD dementia. **eTable 14.** Summary of model selection approach for predictors (plasma and imaging) using longitudinal tau PET as outcome in all CU and MCI independent of Aβ status. **eTable 15.** Power enrichment analysis of plasma biomarkers in a theoretical clinical trial using tau PET as end point in all CU and MCI independent of Aβ status. **eTable 16.** Summary of model selection approach for predictors (plasma and imaging) using longitudinal tau PET as outcome in AD dementia. **eTable 17.** Power enrichment analysis of plasma biomarkers in a theoretical clinical trial using tau PET as end point in AD dementia. **eTable 18.** BioFINDER-1 participant characteristics including A/T/N biomarkers and longitudinal tau PET. **eTable 19.** Relationship between individual biomarkers and tau PET using EBM ROIs and plasma biomarkers in Aβ-positive CU and Aβ-positive MCI from BioFINDER-1. **eTable 20.** Relationship between individual biomarkers and tau PET using EBM ROIs and CSF biomarkers in Aβ-positive CU and Aβ-positive MCI from BioFINDER-1.
eReferences.
