# Supplementary material for: Biomarker-Based Prediction of Longitudinal Tau Positron Emission Tomography in Alzheimer Disease
Source: JAMA Neurol. 2021 Dec 20;79(2):1–11. doi: 10.1001/jamaneurol.2021.4654 (PMC8689441; doi:10.1001/jamaneurol.2021.4654)
Supplement: Supplement. — eMethods 1. Inclusion and exclusion criteria for the Swedish BioFINDER-2 study. eMethods 2. Inclusion and exclusion criteria for the Swedish BioFINDER-1 study. eFigure. Overview of the steps involved in the clustering and EBM derived ROIs. eTable 1. Participant characteristics including A/T/N biomarkers and longitudinal. tau PET in Aβ-negative MCI and CU and MCI regardless of Aβ status. eTable 2. Relationship between individual biomarkers and tau PET using EBM ROIs and plasma biomarkers in all CU and MCI independent of Aβ status. eTable 3. Relationship between individual biomarkers and tau PET using EBM ROIs and CSF biomarkers in all CU and MCI independent of Aβ status. eTable 4. Relationship between individual biomarkers and tau PET using CSF biomarkers. eTable 5. Relationship between individual biomarkers and tau PET using EBM ROIs and CSF biomarkers in AD dementia. eTable 6. Characteristics of Aβ-negative CU individuals used for z-score transformation. eTable 7. Range of annual percent change values and results of one-sample t-tests across groups and EBM ROIs. eTable 8. Mean annual percent change in tau PET SUVR using Braak ROIs. eTable 9. Relationship between individual biomarkers and tau PET using plasma p-tau181. eTable 10. Relationship between individual biomarkers and tau PET using CSF p-tau181. eTable 11. Relationship between individual biomarkers and tau PET using Braak I ROI for Aβ-positive CU and Braak III ROI in Aβ-positive MCI. eTable 12. Relationship between individual biomarkers and tau PET in Aβ-positive CU using transentorhinal SUVR at baseline instead of EBM I baseline SUVR. eTable 13. Relationship between individual biomarkers and tau PET using EBM ROIs and plasma biomarkers in AD dementia. eTable 14. Summary of model selection approach for predictors (plasma and imaging) using longitudinal tau PET as outcome in all CU and MCI independent of Aβ status. eTable 15. Power enrichment analysis of plasma biomarkers in a theoretical clinical trial using [file jamaneurol-e214654-s001.pdf]

Supplemental Online Content

Leuzy A, Smith R, Cullen NC, et al. Biomarker-based prediction of longitudinal tau positron emission tomography in Alzheimer disease. *JAMA Neurol*. Published online December 20, 2021. doi:10.1001/jamaneurol.2021.4654

eMethods 1. Inclusion and exclusion criteria for the Swedish BioFINDER-2 study
eMethods 2. Inclusion and exclusion criteria for the Swedish BioFINDER-1 study
eFigure. Overview of the steps involved in the clustering and EBM derived ROIs
eTable 1. Participant characteristics including A/T/N biomarkers and longitudinal tau PET in Aβ-negative MCI and CU and MCI regardless of Aβ status
eTable 2. Relationship between individual biomarkers and tau PET using EBM ROIs and plasma biomarkers in all CU and MCI independent of Aβ status
eTable 3. Relationship between individual biomarkers and tau PET using EBM ROIs and CSF biomarkers in all CU and MCI independent of Aβ status
eTable 4. Relationship between individual biomarkers and tau PET using CSF biomarkers
eTable 5. Relationship between individual biomarkers and tau PET using EBM ROIs and CSF biomarkers in AD dementia
eTable 6. Characteristics of Aβ-negative CU individuals used for z-score transformation
eTable 7. Range of annual percent change values and results of one-sample t-tests across groups and EBM ROIs
eTable 8. Mean annual percent change in tau PET SUVR using Braak ROIs
eTable 9. Relationship between individual biomarkers and tau PET using plasma p-tau181
eTable 10. Relationship between individual biomarkers and tau PET using CSF p-tau181
eTable 11. Relationship between individual biomarkers and tau PET using Braak I ROI for Aβ-positive CU and Braak III ROI in Aβ-positive MCI
eTable 12. Relationship between individual biomarkers and tau PET in Aβ-positive CU using transentorhinal SUVR at baseline instead of EBM I baseline SUVR
eTable 13. Relationship between individual biomarkers and tau PET using EBM ROIs and plasma biomarkers in AD dementia
eTable 14. Summary of model selection approach for predictors (plasma and imaging) using longitudinal tau PET as outcome in all CU and MCI independent of Aβ status
eTable 15. Power enrichment analysis of plasma biomarkers in a theoretical clinical trial using tau PET as end point in all CU and MCI independent of Aβ status
eTable 16. Summary of model selection approach for predictors (plasma and imaging) using longitudinal tau PET as outcome in AD dementia
eTable 17. Power enrichment analysis of plasma biomarkers in a theoretical clinical trial using tau PET as end point in AD dementia
eTable 18. BioFINDER-1 participant characteristics including A/T/N biomarkers and longitudinal tau PET

© 2021 Leuzy A et al. *JAMA Neurology*.

eTable 19. Relationship between individual biomarkers and tau PET using EBM ROIs and plasma biomarkers in Aβ-positive CU and Aβ-positive MCI from BioFINDER-1
eTable 20. Relationship between individual biomarkers and tau PET using EBM ROIs and CSF biomarkers in Aβ-positive CU and Aβ-positive MCI from BioFINDER-1
eReferences.

This supplemental material has been provided by the authors to give readers additional information about their work.

© 2021 Leuzy A et al. *JAMA Neurology*.

eMethods

eMethods 1. Inclusion and exclusion criteria for the Swedish BioFINDER-2 study

The BioFINDER-2 study enrolls participants in five sub-cohorts; Cohort A and B includes neurologically and cognitively healthy controls. The inclusion criteria are: i) ages 40-65 years (cohort A) and ages 66-100 years (cohort B); ii) absence of cognitive symptoms as assessed by a physician with special interest in cognitive disorders; iii) MMSE score 27-30 (A) or 26-30 (cohort B) at screening visit; iv) do not fulfill the criteria for MCI or any dementia according to DSM-5[1] v) fluent in Swedish. The recruitment process of cohorts A and B is designed to build two study populations with 50% *APOE* ε4 carriers in each.

Cohort C comprises participants with subjective cognitive deficits (SCD) or minor neurocognitive impairment (MCI) (the latter according to DSM-5.[1] Inclusion criteria are: i) Age 40-100 years; ii) referred to the memory clinics due to cognitive symptoms; iii) MMSE score of 24 – 30 points; iv) does not fulfill the criteria for any dementia (major neurocognitive disorder) according to DSM-5[1], v) fluent in Swedish. In accordance with the research framework by the National Institute on Aging-Alzheimer's Association[2] study participants with SCD were analyzed together with the cognitively healthy participants (and combined in the cognitively unimpaired group). Participants were classified as having MCI if they performed worse than -1.5 SD in any cognitive domain according to age and education stratified test norms. The neuropsychological battery covered the domains attention/executive function (Trail Making Test A and B, Symbol Digit Modalities Test, and AQT), memory (10 word immediate and delayed recall from the Alzheimer's Disease Assessment Scale [ADAS]), verbal ability (verbal fluency and the short version of the Boston Naming Test) and visuospatial function (incomplete letters and cube analysis from the Visual Object and Space Perception battery). Those that were not classified as MCI were considered to have SCD.

© 2021 Leuzy A et al. *JAMA Neurology*.

Cohort D consists of participants with dementia due to AD. Inclusion criteria are: i) Age 40-100 years; ii) referred to the memory clinics due to cognitive symptoms; iii) MMSE score of ≥12 points; iv) fulfill the DSM-5 criteria for dementia (major neurocognitive disorder) due to Alzheimer's disease[1]; v) fluent in Swedish.

Exclusion criteria for all sub-cohorts are: i) significant unstable systemic illness that makes it difficult to participate in the study; ii) current significant alcohol or substance misuse; iii) refusing lumbar puncture, MRI or PET.

© 2021 Leuzy A et al. *JAMA Neurology*.

eMethods 2. Inclusion and exclusion criteria for the Swedish BioFINDER-1 study

The healthy elderly participants without cognitive symptoms (n=318) were originally enrolled from the population-based EPIC cohort.[3] The inclusion criteria were (1) age ≥60 years old, (2) Mini-Mental State Examination (MMSE) score of 28-30 points, and (3) fluent in Swedish. The exclusion criteria were (1) presence of subjective cognitive impairment, (2) significant neurologic disease (for example, stroke, Parkinson's disease, multiple sclerosis), (3) severe psychiatric disease (for example, severe depression or psychotic syndromes), and (4) dementia or mild cognitive impairment (MCI).

The inclusion criteria for patients with subjective cognitive decline (SCD) or MCI were that they (1) were referred to participating memory clinics because of cognitive complaints; (2) did not fulfill the criteria for dementia; (3) had a MMSE score of 24 to 30 points; (4) were aged 60 to 80 years; and (5) were fluent in Swedish. The exclusion criteria were (1) cognitive impairment that without doubt could be explained by another condition (other than prodromal dementia), such as brain tumor, brain trauma etc.; (2) severe somatic disease; and (3) refusing lumbar puncture or neuropsychological testing.

The patients with Alzheimer's disease (AD) dementia fulfilled the NIA-AA criteria for probable AD[4] and in the present study we also required that all were Aβ positive. In agreement with the latest research criteria for AD[2], the healthy elderly participants and patients with SCD were classified as cognitively unimpaired.

© 2021 Leuzy A et al. *JAMA Neurology*.

eFigures

eFigure 1. Overview of the steps involved in the clustering and EBM derived ROIs

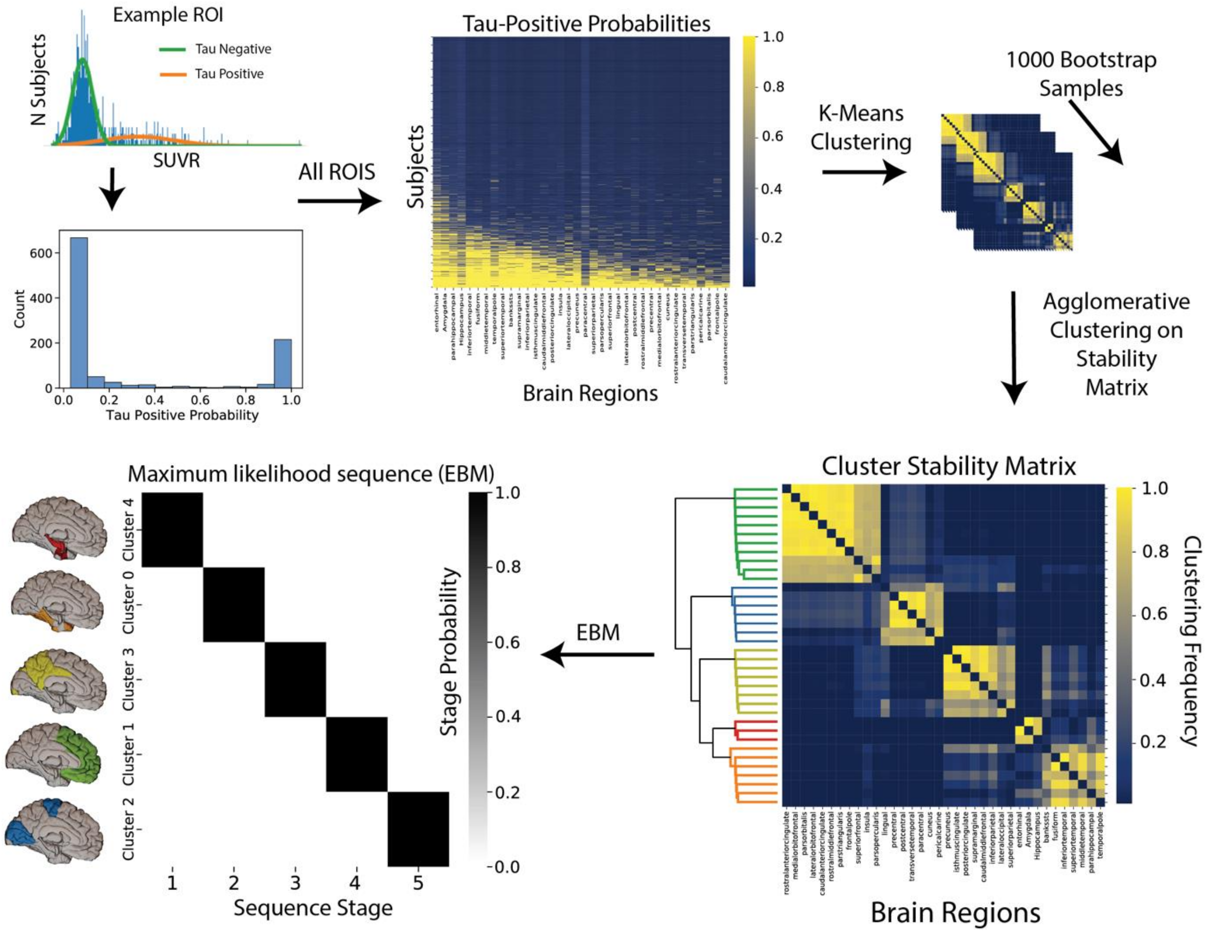

In order to generate the regions of interest used in the primary analyses, a two-component Gaussian mixture model was first applied to all available baseline [^{18}F]RO948 SUVR data (cognitively unimpaired, n=464; mild cognitive impairment, n=196; AD dementia, n=150; non-AD disorders, n=216). For each FreeSurfer ROI, the SUVR value was converted to a tau-positive probability (i.e., the probability that a subjects' SUVR within that ROI value fell within the rightmost portion of the Gaussian distribution, representing abnormal signal). Recursive k-means clustering (n=1000) was then performed on the bootstrapped samples of the input probability data. After each clustering iteration, information about cluster membership is stored in the form of an adjacency matrix. The adjacency matrices were

© 2021 Leuzy A et al. *JAMA Neurology*.

averaged resulting in a stability matrix representing probabilities of different regions clustering together. Lastly, hierarchical agglomerative clustering with Ward criterion was applied to the stability matrix in order to obtain the final clustering solution, with five-cluster solution adopted based on silhouette scores. Identified clusters were then submitted to an event-based model (EBM) to determine in which order the identified clusters become abnormal. In EBM, an 'event' represents the transition from a normal to an abnormal state, with the EBM determining the event sequence that maximizes the data likelihood (i.e., the most likely ordering of the events). Regional tau-positive probabilities were averaged within cluster-derived ROIs and submitted to EBM, using 10,000 Monte-Carlo simulations to derivate uncertainty in event ordering.

© 2021 Leuzy A et al. *JAMA Neurology*.

eTables

eTable 1. Participant characteristics including A/T/N biomarkers and longitudinal tau PET in Aβ negative MCI and CU and MCI regardless of Aβ status

	Aβ- MCI	CU	MCI
N	36	186	94
Age, years	70.52 (8.28)	70.63 (7.24)	70.80 (8.28)
Sex, n (% female)	16 (44%)	90 (48%)	48 (51%)
Education, years	12.53 (3.60)	12.81 (3.39)	12.90 (4.76)
MMSE	27.72 (1.99)	28.91 (1.26)	27.15 (2.01)
APOE ε4 carrier, n (%)	8 (22%)	93 (50%)	45 (48%)
Tau PET, scan interval, years	1.73 (0.16)	1.77 (0.18)	1.71 (0.21)
Baseline tau PET SUVR			
EBM stage I	0.98 (0.14)	0.98 (0.18)	1.20 (0.33)
EBM stage II	1.27 (0.09)	1.26 (0.15)	1.45 (0.33)
EBM stage III	1.23 (0.10)	1.24 (0.16)	1.40 (0.40)
EBM stage IV	1.12 (0.09)	1.11 (0.11)	1.16 (0.14)
EBM stage V	1.16 (0.10)	1.16 (0.11)	1.18 (0.13)
% change tau PET SUVR			
EBM stage I	1.80 [0.76, 2.84]	1.31 [0.79, 1.83]	3.14 [2.37, 3.91]
EBM stage II	0.44 [-0.06, 0.94]	0.65 [0.36, 0.94]	2.47 [1.70, 3.24]
EBM stage III	0.51 [-0.15, 1.17]	0.26 [-0.09, 0.61]	2.58 [1.74, 3.42]
EBM stage IV	0.56 [-0.14, 1.30]	0.33 [-0.09, 0.76]	1.63 [0.99, 2.26]
EBM stage V	0.58 [-0.10, 1.18]	0.34 [-0.04, 0.72]	1.72 [1.05, 2.39]
A/T/N Predictors			
Plasma Aβ42/40	0.21 (0.04)	0.22 (0.05)	0.20 (0.03)
Amyloid PET, Centiloids	-9.33 (9.47)	2.38 (26.94)	33.43 (41.25)

© 2021 Leuzy A et al. *JAMA Neurology*.

Plasma p-tau217, pg/mL	1.64 (6.11)	1.33 (2.20)	2.97 (4.87)
CSF Aβ42/40	0.10 (0.02)	0.09 (0.03)	0.07 (0.03)
CSF p-tau217, pg/mL	63.44 (39.98)	80.68 (93.45)	187.22 (162.81)
Plasma NfL, pg/mL	16.60 (8.86)	12.86 (6.64)	19.95 (11.41)
CSF NfL, pg/mL	194.08 (117.18)	134.59 (92.95)	214.87 (136.72)
Hippocampal volume, mm^3	3378.22 (680.06)	3810.95 (478.14)	3265.31 (590.28)
AD cortex thickness, mm	2.64 (0.18)	2.74 (0.13)	2.60 (0.18)

CU, cognitively unimpaired; MCI, mild cognitive impairment; Aβ42/40, ratio of Aβ42 to Aβ40; PET, positron emission tomography; p-tau217, tau phosphorylated at threonine 217; NfL, neurofilament light; AD cortex, cortical thickness in a temporal meta-ROI encompassing temporal regions with known susceptibility in AD; Amyloid PET refers to the SUVR at baseline in a neocortical meta-ROI, expressed in Centiloids. Stage I = amygdala, hippocampus, entorhinal cortex; Stage II = banks of superior temporal sulcus, fusiform gyrus, inferior temporal cortex, middle temporal gyrus, parahippocampus, superior temporal cortex, temporal pole; Stage III = caudal middle frontal cortex, inferior parietal cortex, isthmus cingulate, lateral occipital cortex, posterior cingulate cortex, precuneus, superior parietal cortex, supramarginal cortex; Stage IV = caudal anteriorcingulate, frontal pole, insula, lateralor bitofrontal cortex, medial orbitofrontal cortex, parsopercularis, parsorbitalis, parstriangularis, rostral anteriorcingulate, rostral middlefrontal cortex, superiorfrontal cortex; Stage V = cuneus, lingual gyrus, paracentral cortex, pericalcarine cortex, postcentral cortex, precentral postcentral cortex, transverse temporal cortex.

© 2021 Leuzy A et al. *JAMA Neurology*.

eTable 2. Relationship between individual biomarkers and tau PET using EBM ROIs and plasma biomarkers in all CU and MCI independent of Aβ status

**Group**	**Predictor**	**Coefficient**	**Adjusted R^2**	**P**	**Δ AIC**
CU	Plasma Aβ42/40	-0.06	0.00 [-0.02, 0.02]	0.4777	1
	Amyloid PET	0.35	0.12 [0.02, 0.22]	<0.0001	-15
	Plasma p-tau217	0.33	0.11 [0.01, 0.21]	0.0001	-13
	Tau PET	0.32	0.10 [-0.07, 0.25]	0.0002	-12
	Plasma NFL	0.16	0.03 [-0.03, 0.08]	0.0669	-1
	Hippocampal volume	-0.03	0.00 [-0.01, 0.01]	0.7266	2
	AD cortex	-0.16	0.03 [-0.03, 0.08]	0.0647	-1
MCI	Plasma Aβ42/40	0.11	0.01 [-0.04, 0.06]	0.3869	1
	Amyloid PET	0.37	0.13 [-0.01, 0.28]	0.0021	-8
	Plasma p-tau217	0.53	0.27 [0.10, 0.45]	<0.0001	-20
	Tau PET	0.59	0.34 [0.17, 0.52]	<0.0001	-26
	Plasma NFL	0.02	0.00 [-0.01, 0.01]	0.8764	2
	Hippocampal volume	-0.18	0.03 [-0.05, 0.11]	0.1554	0
	AD cortex	-0.10	0.01 [-0.03, 0.05]	0.4302	1

CU, cognitively unimpaired; MCI, mild cognitive impairment; Aβ42/40, ratio of Aβ42 to Aβ40; PET, positron emission tomography; p-tau217, tau phosphorylated at threonine 217; NfL, neurofilament light; AD cortex, cortical thickness in a temporal meta-ROI encompassing temporal regions with known susceptibility in AD; R^2, coefficient of determination; P, p-value; Δ AIC, change in Akaike information criterion relative to a demographics only model (age and sex). In CU, tau PET refers to [^{18}F]RO948 SUVR at baseline in the EBM stage I ROI; in MCI, tau PET refers to [^{18}F]RO948 SUVR at baseline in the EBM stage II ROI. Amyloid PET refers to the SUVR at baseline in a neocortical meta-ROI, expressed in Centiloids.

© 2021 Leuzy A et al. *JAMA Neurology*.

eTable 3. Relationship between individual biomarkers and tau PET using EBM ROIs and CSF biomarkers in all CU and MCI independent of Aβ status

Group	Predictor	Coefficient	Adjusted R^2	P	Δ AIC
CU	CSF Aβ42/40	-0.20	0.04 [-0.02, 0.10]	0.0212	-3
	Amyloid PET	0.35	0.12 [0.02, 0.22]	<0.0001	-15
	CSF p-tau217	0.46	0.21 [0.09, 0.35]	0.0001	-30
	Tau PET	0.32	0.10 [-0.07, 0.25]	0.0002	-12
	CSF NFL	0.05	0.00 [-0.02, 0.02]	0.5371	2
	Hippocampal volume	-0.03	0.00 [-0.01, 0.01]	0.7266	2
	AD cortex	-0.16	0.03 [-0.03, 0.08]	0.0647	-1
MCI	CSF Aβ42/40	-0.20	0.04 [-0.05, 0.13]	0.1009	-1
	Amyloid PET	0.37	0.13 [-0.01, 0.28]	0.0021	-8
	CSF p-tau217	0.63	0.39 [0.22, 0.56]	<0.0001	-32
	Tau PET	0.59	0.34 [0.17, 0.52]	<0.0001	-26
	CSF NFL	0.08	0.01 [-0.03, 0.04]	0.5010	2
	Hippocampal volume	-0.18	0.03 [-0.05, 0.11]	0.1554	0
	AD cortex	-0.10	0.01 [-0.03, 0.05]	0.4302	1

CU, cognitively unimpaired; MCI, mild cognitive impairment; Aβ42/40, ratio of Aβ42 to Aβ40; PET, positron emission tomography; p-tau217, tau phosphorylated at threonine 217; NfL, neurofilament light; AD cortex, cortical thickness in a temporal meta-ROI encompassing temporal regions with known susceptibility in AD; R^2, coefficient of determination; P, p-value; Δ AIC, change in Akaike information criterion relative to a demographics only model (age and sex). In Aβ+ CU, tau PET refers to [^{18}F]RO948 SUVR at baseline in the EBM stage I ROI; in Aβ+ MCI, tau PET refers to [^{18}F]RO948 SUVR at baseline in the EBM stage II ROI. Amyloid PET refers to the SUVR at baseline in a neocortical meta-ROI, expressed in Centiloids.

© 2021 Leuzy A et al. *JAMA Neurology*.

eTable 4. Relationship between individual biomarkers and tau PET using CSF biomarkers

**Group**	**Predictor**	**Coefficient**	**Adjusted R^2**	**P**	**Δ AIC**
Aβ+ CU	CSF Aβ42/40	0.13	0.02 [-0.06, 0.09]	0.4402	1
	Amyloid PET	0.32	0.10 [-0.07, 0.27]	0.0571	-2
	CSF p-tau217	0.60	0.35 [0.12, 0.58]	0.0001	-14
	Tau PET	0.36	0.13 [-0.06, 0.31]	0.0294	-3
	CSF NfL	0.05	0.00 [-0.03 0.03]	0.7660	2
	Hippocampal volume	0.06	0.00 [-0.03, 0.04]	0.7234	2
	AD cortex	-0.07	0.01 [-0.04, 0.05]	0.6707	2
Aβ+ MCI	CSF Aβ42/40	-0.03	0.00 [-0.02, 0.02]	0.8272	2
	Amyloid PET	0.16	0.02 [-0.06, 0.11]	0.3035	1
	CSF p-tau217	0.52	0.27 [0.06, 0.47]	0.0002	-12
	Tau PET	0.67	0.44 [0.16, 0.57]	<0.0001	-25
	CSF NfL	0.08	0.01 [-0.04, 0.05]	0.6022	2
	Hippocampal volume	-0.18	0.03 [-0.06, 0.13]	0.2335	0
	AD cortex	-0.16	0.02 [-0.06, 0.11]	0.3051	1

Aβ+, amyloid-β positive; CU, cognitively unimpaired; MCI, mild cognitive impairment; CSF, cerebrospinal fluid; Aβ42/40, ratio of Aβ42 to Aβ40; PET, positron emission tomography; p-tau217, tau phosphorylated at threonine 217; NfL, neurofilament light; AD cortex, cortical thickness in a temporal meta-ROI encompassing temporal regions with known susceptibility in AD; R^2, coefficient of determination; P, p-value; Δ AIC, change in Akaike information criterion relative to a demographics only model (age and sex). In Aβ+ CU, tau PET refers to [^{18}F]RO948 SUVR at baseline in the EBM stage I ROI; in Aβ+ MCI, tau PET refers to [^{18}F]RO948 SUVR at baseline in the EBM stage II ROI. Amyloid PET refers to the SUVR at baseline in a neocortical meta ROI, expressed in Centiloids.

© 2021 Leuzy A et al. *JAMA Neurology*.

eTable 5. Relationship between individual biomarkers and tau PET using EBM ROIs and CSF biomarkers in AD dementia

Group	Predictor	Coefficient	Adjusted R^2	P	Δ AIC
AD dementia	CSF Aβ42/40	0.21	0.04 [-0.06, 0.15]	0.1482	0
	CSF p-tau217	0.48	0.23 [0.03, 0.42]	0.0005	-11
	Tau PET	0.55	0.29 [0.09, 0.49]	0.0001	-15
	CSF NFL	-0.13	0.02 [-0.05, 0.08]	0.3700	1
	Hippocampal volume	-0.011	0.01 [-0.04, 0.07]	0.4688	1
	AD cortex	0.10	0.01 [-0.04, 0.06]	0.5163	2

AD, Alzheimer disease; Aβ42/40, ratio of Aβ42 to Aβ40; PET, positron emission tomography; p-tau217, tau phosphorylated at threonine 217; NfL, neurofilament light; AD cortex, cortical thickness in a temporal meta ROI encompassing temporal regions with known susceptibility in AD; R^2, coefficient of determination; P, p value; Δ AIC, change in Akaike information criterion relative to a demographics only model (age and sex). Tau PET refers to [^{18}F]RO948 SUVR at baseline in the EBM stage IV ROI. In BioFINDER-2, amyloid PET is by design performed only in individuals without cognitive impairment and those with MCI.

© 2021 Leuzy A et al. *JAMA Neurology*.

eTable 6. Characteristics of Aβ-negative CU individuals used for z-score transformation

	Aβ- CU
N	61
Age, years	51.85 (3.41)
Sex, n (% female)	33 (54%)
Education, years	13.62 (2.53)
MMSE	28.97 (1.19)
APOE ε4 carrier, n (%)	25 (41%)
Tau PET, scan interval, years	1.76 (0.17)
EBM stage I, baseline SUVR	0.93 (0.20)
EBM stage II, baseline SUVR	1.23 (0.09)
EBM stage I, % annual Δ SUVR	0.21 (2.07)
EBM stage II, % annual Δ SUVR	0.24 (1.51)
Plasma Aβ42/40	0.25 (0.04)
CSF Aβ42/40, pg/mL	0.11 (0.01)
Amyloid PET, Centiloids	-9.80 (5.50)
Plasma p-tau217, pg/mL	0.95 (0.73)
CSF p-tau217, pg/mL	54.89 (48.83)
Plasma NfL, pg/mL	9.56 (3.69)
CSF NfL, pg/mL	103.39 (29.52)
Hippocampal volume, mm^3	3917 (464.63)
AD cortex, mm	2.78 (0.15)

Aβ-, amyloid-β negative; CU, cognitively unimpaired; MMSE, mini mental state examination score; APOE, apolipoprotein E; PET, positron emission tomography; EBM, event-based modelling; SUVR, standardized uptake value ratio; Aβ42/40, ratio of Aβ42 to Aβ40; CSF, cerebrospinal fluid; p-tau217, tau phosphorylated at threonine 217; NfL, neurofilament light; AD cortex, cortical thickness in a

© 2021 Leuzy A et al. *JAMA Neurology*.

temporal meta-ROI encompassing temporal regions with known susceptibility in AD. Amyloid PET refers to the SUVR at baseline in a neocortical meta-ROI, expressed in Centiloids; Stage I = amygdala, hippocampus, entorhinal cortex; Stage II = banks of superior temporal sulcus, fusiform gyrus, inferior temporal cortex, middle temporal gyrus, parahippocampus, superior temporal cortex, temporal pole;

© 2021 Leuzy A et al. *JAMA Neurology*.

eTable 7. Range of annual percent change values and results of one-sample t-tests across groups and EBM ROIs

	Range (min, max); Significance level from one sample t-test				
EBM ROI	Aβ- CU	Aβ+ CU	Aβ- MCI	Aβ+ MCI	AD dementia
Stage I	-2.04, 7.12; P<0.001	-6.70, 12.60; P<0.0001	-3.40, 5.06; P<0.001	-4.81, 10.21; P<0.0001	-3.42, 13.19; P<0.0001
Stage II	-6.59, 4.61; P=0.267	-3.89, 7.60; P<0.0001	-3.01, 3.22; P=0.107	-3.45, 17.49; P<0.0001	-4.79, 16.01; P<0.0001
Stage III	-3.50, 4.65; P=0.395	-2.93, 5.51; P<0.0001	-4.69, 4.99; P=0.163	-3.87, 8.59; P<0.0001	-2.59, 16.11; P<0.0001
Stage IV	-5.10, 3.65; P=410	-2.13, 4.15; P<0.0001	-4.30, 4.60; P=0.141	-2.49, 6.56; P<0.0001	-7.46, 16.24; P<0.0001
Stage V	-3.69, 4.61; P=0.492	-2.29, 3.53; P<0.001	-2.57, 3.29; P=0.228	-3.16, 6.62; P<0.0001	-3.87, 15.17; P<0.0001

Mininum and maximum annual percent change is shown along with p-values from one sample t-tests showing whether change was significantly different from zero. EBM, event-based modelling; ROI, region of interest; Aβ-, amyloid-β negative; Aβ+, amyloid-β positive; CU, cognitively unimpaired; MCI, mild cognitive impairment; AD, Alzheimer disease. Stage I = amygdala, hippocampus, entorhinal cortex; Stage II = banks of superior temporal sulcus, fusiform gyrus, inferior temporal cortex, middle temporal gyrus, parahippocampus, superior temporal cortex, temporal pole; Stage III = caudal middle frontal cortex, inferior parietal cortex, isthmus cingulate, lateral occipital cortex, posterior cingulate cortex, precuneus, superior parietal cortex, supramarginal cortex; Stage IV = caudal anteriorcingulate, frontal pole, insula, lateralor bitofrontal cortex, medial orbitofrontal cortex, parsopercularis, parsorbitalis, parstriangularis, rostral anteriorcingulate, rostral middlefrontal cortex, superiorfrontal cortex; Stage V = cuneus, lingual gyrus, paracentral cortex, pericalcarine cortex, postcentral cortex, precentral postcentral cortex, transverse temporal cortex.

© 2021 Leuzy A et al. *JAMA Neurology*.

eTable 8. Mean annual percent change in tau PET SUVR using Braak ROIs

	Mean annual % change [95% CI]						
ROI	Aβ- CU	Aβ+ CU	All CU	Aβ- MCI	Aβ+ MCI	All MCI	AD dementia
Braak I	0.90 [0.11, 1.69]	2.80 [0.84, 4.76]	1.20 [0.46, 1.94]	0.69 [-0.92, 2.30]	3.90 [3.10, 4.80]	1.96 [1.07, 2.85]	2.38 [0.74, 4.02]
Braak II	1.10 [0.19, 2.00]	5.14 [1.92, 7.21]	1.75 [0.81, 2.69]	2.01 [0.66, 3.60]	3.50 [2.34, 4.66]	2.49 [1.58, 3.40]	2.98 [1.32, 4.64]
Braak III	0.60 [0.09, 1.11]	2.71 [1.78, 3.64]	0.94 [0.45, 1.43]	0.54 [-0.25, 1.33]	4.67 [3.68, 5.86]	2.85 [2.06, 3.64]	3.93 [2.60, 5.26]
Braak IV	0.25 [-0.09, 0.59]	2.32 [1.62, 3.02]	0.58 [0.23, 0.93]	0.53 [-0.22, 1.28]	4.05 [2.76, 5.08]	2.18 [1.21, 3.15]	5.04 [3.72, 6.36]
Braak V	0.24 [-0.15, 0.63]	2.33 [1.59, 3.07]	0.58 [0.20, 0.97]	0.52 [-0.07, 1.13]	1.96 [1.07, 2.84]	2.12 [1.39, 2.85]	5.12 [3.89, 6.35]
Braak VI	0.20 [-0.25, 0.65]	2.30 [1.36, 3.24]	0.54 [0.13, 1.03]	0.56 [-0.18, 1.29]	1.60 [0.71, 2.49]	1.69 [0.96, 2.42]	3.98 [2.81, 5.15]

ROI, region of interest; Aβ-, amyloid-β negative; Aβ+, amyloid-β positive; CU, cognitively unimpaired; MCI, mild cognitive impairment; AD, Alzheimer's disease. Braak I = entorhinal cortex; Braak II = hippocampus; Braak III = amygdala, fusiform gyrus, parahippocampus; Braak IV, inferior temporal, middle temporal cortex; Braak V = caudal anterior cingulate, caudal middle frontal, frontal pole, inferior parietal, insula, lateral occipital, lateral orbitofrontal, lingual, medial orbitofrontal, posterior cingulate, precuneus, rostralanteriorcingulate, rostralmiddlefrontal, superiorfrontal, superiorparietal, superiortemporal, supramarginal gyrus; Braak VI = Cuneus, paracentral, pericalrine, postcentral, precentral gyrus a s

© 2021 Leuzy A et al. *JAMA Neurology*.

eTable 9. Relationship between individual biomarkers and tau PET using plasma p-tau181

Group	Predictor	Coefficient	Adjusted R^2	P	Δ AIC
Aβ+ CU	Plasma Aβ42/40	-0.03	0.00 [-0.02, 0.02]	0.8615	2
	Amyloid PET	0.32	0.10 [-0.07, 0.27]	0.0471	-2
	Plasma p-tau181	0.42	0.17 [-0.03, 0.37]	0.0113	-5
	Tau PET	0.36	0.13 [-0.06, 0.31]	0.0294	-3
	Plasma NFL	0.10	0.01 [-0.05, 0.07]	0.5664	2
	Hippocampal volume	0.06	0.00 [-0.03, 0.04]	0.7234	2
	AD cortex	-0.07	0.01 [-0.04, 0.05]	0.6707	2
Aβ+ MCI	Plasma Aβ42/40	-0.24	0.05 [-0.06, 0.17]	0.1190	-1
	Amyloid PET	0.16	0.02 [-0.06, 0.11]	0.3035	1
	Plasma p-tau181	0.41	0.16 [-0.02, 0.34]	0.0051	-6
	Tau PET	0.67	0.44 [0.16, 0.57]	<0.0001	-25
	Plasma NFL	-0.07	0.00 [-0.03, 0.04]	0.6702	2
	Hippocampal volume	-0.18	0.03 [-0.06, 0.13]	0.2335	0
	AD cortex	-0.16	0.02 [-0.06, 0.11]	0.3051	1

Aβ+, amyloid-β positive; CU, cognitively unimpaired; MCI, mild cognitive impairment; Aβ42/40, ratio of Aβ42 to Aβ40; PET, positron emission tomography; p-tau181, tau phosphorylated at threonine 181; NfL, neurofilament light; AD cortex, cortical thickness in a temporal meta-ROI encompassing temporal regions with known susceptibility in AD; R^2, coefficient of determination; P, p-value; Δ AIC, change in Akaike information criterion relative to a demographics only model (age and sex). In Aβ+ CU, tau PET refers to [^{18}F]RO948 SUVR at baseline in the Braak I ROI; in Aβ+ MCI, tau PET refers to [^{18}F]RO948 SUVR at baseline in the Braak II ROI. Amyloid PET refers to the SUVR at baseline in a neocortical meta-ROI, expressed in Centiloids.

© 2021 Leuzy A et al. *JAMA Neurology*.

eTable 10. Relationship between individual biomarkers and tau PET using CSF p-tau181

Group	Predictor	Coefficient	Adjusted R^2	P	Δ AIC
Aβ+ CU	CSF Aβ42/40	0.13	0.02 [-0.06, 0.09]	0.4402	1
	Amyloid PET	0.32	0.10 [-0.07, 0.27]	0.0571	-2
	CSF p-tau181	0.51	0.25 [0.03, 0.48]	0.0014	-9
	Tau PET	0.36	0.13 [-0.06, 0.31]	0.0294	-3
	CSF NfL	0.05	0.00 [-0.03 0.03]	0.7660	2
	Hippocampal volume	0.06	0.00 [-0.03, 0.04]	0.7234	2
	AD cortex	-0.07	0.01 [-0.04, 0.05]	0.6707	2
Aβ+ MCI	CSF Aβ42/40	-0.03	0.00 [-0.02, 0.02]	0.8272	2
	Amyloid PET	0.16	0.02 [-0.06, 0.11]	0.3035	1
	CSF p-tau181	0.46	0.21 [0.02, 0.40]	0.0012	-9
	Tau PET	0.67	0.44 [0.16, 0.57]	<0.0001	-25
	CSF NfL	0.08	0.01 [-0.04, 0.05]	0.6022	2
	Hippocampal volume	-0.18	0.03 [-0.06, 0.13]	0.2335	0
	AD cortex	-0.16	0.02 [-0.06, 0.11]	0.3051	1

Aβ+, amyloid-β positive; CU, cognitively unimpaired; MCI, mild cognitive impairment; Aβ42/40, ratio of Aβ42 to Aβ40; PET, positron emission tomography; p-tau181, tau phosphorylated at threonine 181; NfL, neurofilament light; AD cortex, cortical thickness in a temporal meta-ROI encompassing temporal regions with known susceptibility in AD; R^2, coefficient of determination; P, p-value; Δ AIC, change in Akaike information criterion relative to a demographics only model (age and sex). In Aβ+ CU, tau PET refers to [^{18}F]RO948 SUVR at baseline in the Braak I ROI; in Aβ+ MCI, tau PET refers to [^{18}F]RO948 SUVR at baseline in the Braak II ROI. Amyloid PET refers to the SUVR at baseline in a neocortical meta-ROI, expressed in Centiloids.

© 2021 Leuzy A et al. *JAMA Neurology*.

eTable 11. Relationship between individual biomarkers and tau PET using Braak I ROI for Aβ-positive CU and Braak III ROI in Aβ-positive MCI

Group	Predictor	Coefficient	Adjusted R^2	P	Δ AIC
Aβ+ CU	Plasma Aβ42/40	0.01	0.00 [-0.01, 0.01]	0.9471	2
	Amyloid PET	0.30	0.09 [-0.07, 0.25]	0.0743	-1
	Plasma p-tau217	0.51	0.25 [0.03, 0.47]	0.0015	-9
	Tau PET	0.31	0.09 [-0.07, 0.25]	0.0707	-2
	Plasma NFL	0.03	0.00 [-0.02, 0.02]	0.8422	2
	Hippocampal volume	0.09	0.01 [-0.05, 0.06]	0.5842	2
	AD cortex	0.07	0.01 [-0.04, 0.05]	0.6736	2
Aβ+ MCI	Plasma Aβ42/40	0.01	0.00 [-0.01, 0.01]	0.9495	2
	Amyloid PET	0.13	0.02 [-0.05, 0.09]	0.3831	1
	Plasma p-tau217	0.46	0.21 [0.00, 0.41]	0.0032	-7
	Tau PET	0.64	0.40 [0.20, 0.61]	<0.0001	-22
	Plasma NFL	0.05	0.00 [-0.02, 0.03]	0.7479	2
	Hippocampal volume	-0.05	0.00 [-0.02, 0.03]	0.7464	2
	AD cortex	-0.03	0.00 [-0.01, 0.01]	0.8657	2

Aβ+, amyloid-β positive; CU, cognitively unimpaired; MCI, mild cognitive impairment; Aβ42/40, ratio of Aβ42 to Aβ40; PET, positron emission tomography; p-tau217, tau phosphorylated at threonine 217; NfL, neurofilament light; AD cortex, cortical thickness in a temporal meta-ROI encompassing temporal regions with known susceptibility in AD; R^2, coefficient of determination; P, p-value; Δ AIC, change in Akaike information criterion relative to a demographics only model (age and sex). In Aβ+ CU, tau PET refers to [^{18}F]RO948 SUVR at baseline in the Braak I ROI; in Aβ+ MCI, tau PET refers to [^{18}F]RO948 SUVR at baseline in the Braak II ROI. Amyloid PET refers to the SUVR at baseline in a neocortical meta-ROI, expressed in Centiloids.

© 2021 Leuzy A et al. *JAMA Neurology*.

eTable 12. Relationship between individual biomarkers and tau PET in Aβ-positive CU using transentorhinal SUVR at baseline instead of EBM I baseline SUVR

Group	Predictor	Coefficient	Adjusted R^2	P	Δ AIC
Aβ+ CU	Plasma Aβ42/40	-0.06	0.00 [-0.04, 0.04]	0.7405	2
	Amyloid PET	0.06	0.00 [-0.03, 0.04]	0.7549	2
	Plasma p-tau217	0.53	0.27 [0.03, 0.51]	0.0028	-8
	Tau PET	0.33	0.11 [-0.07, 0.28]	0.0501	-2
	Plasma NFL	0.00	0.00 [-0.00, 0.00]	0.9942	2
	Hippocampal volume	0.15	0.02 [-0.07, 0.11]	0.4410	1
	AD cortex	-0.05	0.00 [-0.03, 0.04]	0.7744	2

Aβ+, amyloid-β positive; CU, cognitively unimpaired; MCI, mild cognitive impairment; Aβ42/40, ratio of Aβ42 to Aβ40; PET, positron emission tomography; p-tau217, tau phosphorylated at threonine 217; NfL, neurofilament light; AD cortex, cortical thickness in a temporal meta-ROI encompassing temporal regions with known susceptibility in AD; R^2, coefficient of determination; P, p-value; Δ AIC, change in Akaike information criterion relative to a demographics only model (age and sex).

© 2021 Leuzy A et al. *JAMA Neurology*.

eTable 13. Relationship between individual biomarkers and tau PET using EBM ROIs and plasma biomarkers in AD dementia

Group	Predictor	Coefficient	Adjusted R^2	P	Δ AIC
AD dementia	Plasma Aβ42/40	0.11	0.01 [-0.05, 0.07]	0.4469	1
	Plasma p-tau217	0.39	0.15 [-0.02, 0.32]	0.0059	-6
	Tau PET	0.55	0.29 [0.09, 0.49]	0.0001	-15
	Plasma NFL	-0.02	0.00 [-0.01, 0.01]	0.9105	2
	Hippocampal volume	-0.011	0.01 [-0.04, 0.07]	0.4688	1
	AD cortex	0.10	0.01 [-0.04, 0.06]	0.5163	2

AD, Alzheimer disease; Aβ42/40, ratio of Aβ42 to Aβ40; PET, positron emission tomography; p-tau217, tau phosphorylated at threonine 217; NfL, neurofilament light; AD cortex, cortical thickness in a temporal meta ROI encompassing temporal regions with known susceptibility in AD; R^2, coefficient of determination; P, p value; Δ AIC, change in Akaike information criterion relative to a demographics only model (age and sex). Tau PET refers to [^{18}F]RO948 SUVR at baseline in the EBM stage IV ROI. In BioFINDER-2, amyloid PET is by design performed only in individuals without cognitive impairment and those with MCI.

© 2021 Leuzy A et al. *JAMA Neurology*.

eTable 14. Summary of model selection approach for predictors (plasma and imaging) using longitudinal tau PET as outcome in all CU and MCI independent of Aβ status

**Group**	**Model**	**Removed from model**	**R^2**	**AIC**
CU	Plasma Aβ42/40, plasma p-tau217, plasma NfL, Hippocampal volume, AD cortex, amyloid PET, tau PET		0.18	363
	Plasma Aβ42/40, plasma p-tau217, Hippocampal volume, AD cortex, amyloid PET, tau PET	Plasma NfL	0.18	361
	Plasma Aβ42/40, plasma p-tau217, Hippocampal volume, amyloid PET, tau PET	AD cortex	0.18	360
	Plasma Aβ42/40, plasma p-tau217, amyloid PET, tau PET	Hippocampal volume	0.18	358
	Plasma Aβ42/40, plasma p-tau217, tau PET	Amyloid PET	0.17	356
	Plasma p-tau217, tau PET	Plasma Aβ42/40	0.17	355
	Plasma p-tau217	Tau PET	0.17	358

© 2021 Leuzy A et al. *JAMA Neurology*.

MCI	Plasma Aβ42/40, plasma p-tau217, plasma NfL, Hippocampal volume, AD cortex, amyloid PET, tau PET		0.42	170
	Plasma Aβ42/40, plasma p-tau217, plasma NfL, Hippocampal volume, AD cortex, tau PET	Amyloid PET	0.42	168
	Plasma Aβ42/40, plasma p-tau217, plasma NfL, AD cortex, tau PET	Hippocampal volume	0.42	167
	Plasma p-tau217, plasma NfL, AD cortex, tau PET	Plasma Aβ42/40	0.42	165
	Plasma p-tau217, AD cortex, tau PET	Plasma NfL	0.41	165
	Plasma p-tau217, tau PET	AD cortex	0.40	165
	Plasma p-tau217	Tau PET	0.38	168

CU, cognitively unimpaired; MCI, mild cognitive impairment; Aβ42/40, ratio of Aβ42 to Aβ40; PET, positron emission tomography; p-tau217, tau phosphorylated at threonine 217; NfL, neurofilament light; AD cortex, cortical thickness in a temporal meta-ROI encompassing temporal regions with known susceptibility in AD; R^2, coefficient of determination; P, p-value; AIC, Akaike information criterion.

In CU, tau PET refers to [^{18}F]RO948 SUVR at baseline in the EBM stage I ROI; in MCI, tau PET refers to [^{18}F]RO948 SUVR at baseline in the EBM stage II ROI.

Amyloid PET refers to the SUVR at baseline in a neocortical meta-ROI, expressed in Centiloids.

© 2021 Leuzy A et al. *JAMA Neurology*.

eTable 15. Power enrichment analysis of plasma biomarkers in a theoretical clinical trial using tau-PET as end point in all CU and MCI independent of Aβ status

Group	Predictor(s)	Sample size reduction (%)	P	Δ AIC
CU	Plasma p-tau217	34 [18, 46%]	<0.01	-12
	Amyloid PET	17% [18, 29%]	<0.001	-8
	Plasma p-tau217 + amyloid PET	43% [29, 57%]	<0.0001	-28
MCI	Plasma p-tau217	42 [34, 61%]	<0.001	-10
	Tau PET	64% [45, 74%]	<0.0001	-18
	Plasma p-tau217 + tau PET	73% [47, 83%]	<0.0001	-28

CU, cognitively unimpaired; MCI, mild cognitive impairment; p-tau217, tau phosphorylated at threonine 217; Amyloid PET refers to the SUVR at baseline in a neocortical meta-ROI, expressed in Centiloids; Tau PET refers to baseline SUVR within the EBM stage-I ROI. Sample size reduction is relative to including all subjects (i.e., no enrichment). 95% confidence intervals are shown in square brackets. P, p-value; Δ AIC, change in Akaike information.

© 2021 Leuzy A et al. *JAMA Neurology*.

eTable 16. Summary of model selection approach for predictors (plasma and imaging) using longitudinal tau PET as outcome in AD dementia

**Group**	**Model**	**Removed from model**	**R^2**	**AIC**
AD dementia	Plasma Aβ42/40, plasma p-tau217, plasma NfL, hippocampal volume, AD cortex, tau PET		0.38	128
	Plasma Aβ42/40, plasma p-tau217, hippocampal volume, AD cortex, tau PET	Plasma NFL	0.38	126
	Plasma Aβ42/40, plasma p-tau217, hippocampal volume, tau PET	AD cortex	0.38	124
	Plasma p-tau217, hippocampal volume, tau PET	Plasma Aβ42/40	0.38	123
	Plasma p-tau217, tau PET	Hippocampal volume	0.37	122
	Tau PET	Plasma p-tau217	0.36	126

AD, Alzheimer disease; Aβ42/40, ratio of Aβ42 to Aβ40; PET, positron emission tomography; p-tau217, tau phosphorylated at threonine 217; NfL, neurofilament light; AD cortex, cortical thickness in a temporal meta-ROI encompassing temporal regions with known susceptibility in AD; R^2, coefficient of determination; P, p value; AIC, Akaike information criterion. Tau PET refers to [^{18}F]RO948 SUVR at baseline in the EBM stage-IV ROI.

© 2021 Leuzy A et al. *JAMA Neurology*.

eTable 17. Power enrichment analysis of plasma biomarkers in a theoretical clinical trial using tau-PET as end point in AD dementia

**Group**	**Predictor(s)**	**Sample size reduction (%)**	**P**	**Δ AIC**
AD dementia	Plasma p-tau217	42% [29-54%]	<0.001	-10
	Tau PET	60% [44-67%]	<0.0001	-18
	Plasma p-tau217 + tau PET	71% [62-84%]	<0.0001	-28

AD, Alzheimer disease; p-tau217, tau phosphorylated at threonine 217; Tau PET refers to baseline SUVR within the EBM stage IV ROI. Sample size reduction is relative to including all subjects (i.e., no enrichment). 95% confidence intervals are shown in square brackets. P, p-value; Δ AIC, change in Akaike information.

© 2021 Leuzy A et al. *JAMA Neurology*.

eTable 18. BioFINDER-1 participant characteristics including A/T/N biomarkers and longitudinal tau PET

	Aβ- CU	Aβ+ CU	Aβ- MCI	Aβ+ MCI	AD dementia
N	12	27	10	16	20
Age, years	74.37 (4.97)	75.33 (5.60)	67.52 (6.54)	69.11 (8.19)	70.55 (8.13)
Sex, n (% female)	7 (58%)	15 (56%)	4 (40%)	8 (50%)	11 (55%)
Education	12.69 (4.45)	12.15 (4.07)	12.59 (2.87)	12.22 (2.86)	12.91 (3.37)
MMSE	28.68 (1.25)	28.93 (1.07)	27.72 (1.99)	26.10 (2.90)	21.38 (4.50)
APOE ε4 carrier, n (%)	3 (25%)	16 (59%)	8 (22%)	11 (69%)	13 (65%)
Tau PET, scan interval, years	1.84 (0.25)	1.89 (0.29)	1.57 (0.26)	1.77 (0.17)	1.87 (0.40)
Baseline tau PET SUVR					
EBM stage I	1.12 (0.08)	1.18 (0.17)	1.10 (0.12)	1.49 (0.18)	1.63 (0.26)
EBM stage II	1.24 (0.06)	1.35 (0.28)	1.24 (0.11)	1.85 (0.57)	2.19 (0.51)
EBM stage III	1.25 (0.07)	1.27 (0.22)	1.21 (0.09)	1.96 (0.91)	2.26 (0.80)
EBM stage IV	1.20 (0.07)	1.17 (0.11)	1.06 (0.10)	1.44 (0.27)	1.62 (0.56)
EBM stage V	1.12 (0.05)	1.15 (0.09)	1.17 (0.03)	1.23 (0.31)	1.50 (0.42)

© 2021 Leuzy A et al. *JAMA Neurology*.

Change tau PET SUVR, %					
EBM stage I	1.39 [1.07, 1.71]	3.44 [2.96, 4.84]	1.65 [0.89, 2.21]	3.47 [3.19, 4.02]	3.08 [2.68, 3.84]
EBM stage II	0.48 [0.27, 0.69]	2.17 [1.77, 2.87]	0.74 [0.18, 1.10]	4.35 [3.92, 4.78]	3.89 [3.24, 4.29]
EBM stage III	0.27 [0.06, 0.48]	1.98 [1.58, 2.58]	0.37 [0.08, 0.68]	3.30 [2.95, 3.64]	4.18 [3.74, 4.87]
EBM stage IV	0.29 [0.01, 0.57]	1.75 [1.35, 2.15]	0.46 [0.23, 0.69]	2.15 [1.90, 2.54]	4.21 [3.82, 4.60]
EBM stage V	0.23 [-0.02, 0.47]	1.11 [0.75, 1.85]	0.49 [0.34, 0.63]	2.26 [1.97, 2.72]	3.39 [3.07, 3.71]
A/T/N Predictors					
Plasma Aβ42/40	0.17 (0.04)	0.12 (0.02)	0.15 (0.03)	0.10 (0.03)	0.07 (0.02)
CSF Aβ42/40, pg/mL	0.09 (0.05)	0.07 (0.03)	0.12 (0.04)	0.05 (0.02)	0.05 (0.01)
Amyloid PET, Centiloids	-6.44 (8.72)	47.54 (19.31)	-9.53 (7.41)	65.43 (21.20)	-[*]
Plasma p-tau181, pg/mL	2.26 (2.98)	3.43 (1.24)	3.09 (5.03)	5.29 (2.39)	5.16 (2.17)
CSF p-tau181, pg/mL	96.75 (31.82)	168.97 (82.90)	139.67 (163.58)	422.17 (172.06)	423.91 (258.73)
Plasma NfL, pg/mL	2.34 (0.89)	3.24 (0.89)	2.96 (0.74)	3.94 (1.12)	4.04 (1.29)
CSF NfL, pg/mL	821.69 (234.59)	1201 (518.09)	1096.62 (737.72)	1604.43 (658.09)	3296.05 (926.30)
Hippocampal volume, mm^3	3564 (357.82)	3394.12 (338.65)	3350.15 (500.81)	3181.93 (336.82)	2885.21 (537.22)
AD cortex thickness, mm	2.65 (0.18)	2.70 (0.14)	2.61 (0.32)	2.49 (0.22)	2.29 (0.23)

© 2021 Leuzy A et al. *JAMA Neurology*.

Aβ-, amyloid-β negative; Aβ+, amyloid-β positive; CU, cognitively unimpaired; MCI, mild cognitive impairment; AD, Alzheimer disease; MMSE, Mini Mental State Examination; APOE, Apolipoprotein E: Tau PET, [^{18}F]flortaucipir PET, EBM, Event Based Modelling; A/T/N, Amyloid/Tau/Neurodegeneration; ratio of Aβ42 to Aβ40; p-tau181, tau phosphorylated at threonine 181; NfL, neurofilament light; AD cortex, cortical thickness in a temporal meta-ROI. Biomarker data is presented as mean (SD) or mean [95% confidence interval]. Stage I = amygdala, hippocampus, entorhinal cortex; Stage II = banks of superior temporal sulcus, fusiform gyrus, inferior temporal cortex, middle temporal gyrus, parahippocampus, superior temporal cortex, temporal pole; Stage III = caudal middle frontal cortex, inferior parietal cortex, isthmus cingulate, lateral occipital cortex, posterior cingulate cortex, precuneus, superior parietal cortex, supramarginal cortex; Stage IV = caudal anteriorcingulate, frontal pole, insula, lateralor bitofrontal cortex, medial orbitofrontal cortex, parsopercularis, parsorbitalis, parstriangularis, rostral anteriorcingulate, rostral middlefrontal cortex, superiorfrontal cortex; Stage V = cuneus, lingual gyrus, paracentral cortex, pericalcarine cortex, postcentral cortex, precentral postcentral cortex, transverse temporal cortex.

© 2021 Leuzy A et al. *JAMA Neurology*.

eTable 19. Relationship between individual biomarkers and tau PET using EBM ROIs and plasma biomarkers in Aβ-positive CU and Aβ-positive MCI from BioFINDER-1

Group	Predictor	Coefficient	Adjusted R^2	P	Δ AIC
Aβ+ CU	Plasma Aβ42/40	-0.10	0.01 [-0.06, 0.08]	0.7026	2
	Amyloid PET	0.36	0.13 [-0.08, 0.33]	0.0416	-2
	Plasma p-tau181	0.47	0.21 [0.09, 0.27]	0.0132	-5
	Tau PET	0.38	0.14 [-0.07, 0.36]	0.0378	-2
	Plasma NFL	0.04	0.00 [-0.03, 0.05]	0.8706	2
	Hippocampal volume	-0.10	0.01 [-0.05, 0.07]	0.6321	2
	AD cortex	0.03	0.00 [-0.02, 0.02]	0.8751	2
Aβ+ MCI	Plasma Aβ42/40	-0.18	0.03 [-0.10, 0.16]	0.4751	1
	Amyloid PET	0.20	0.04 [-0.10, 0.18]	0.4260	1
	Plasma p-tau181	0.42	0.16 [-0.09 0.42]	0.0386	-2
	Tau PET	0.59	0.33 [0.04, 0.62]	0.0094	-6
	Plasma NFL	0.04	0.00 [-0.04, 0.04]	0.7412	2
	Hippocampal volume	-0.16	0.02 [-0.09, 0.14]	0.5299	2
	AD cortex	-0.13	0.02 [-0.08, 0.11]	0.6142	2

Aβ+, amyloid-β positive; CU, cognitively unimpaired; MCI, mild cognitive impairment; Aβ42/40, ratio of Aβ42 to Aβ40; PET, positron emission tomography; p-tau217, tau phosphorylated at threonine 217; NfL, neurofilament light; AD cortex, cortical thickness in a temporal meta-ROI encompassing temporal regions with known susceptibility in AD; R^2, coefficient of determination; P, p-value; Δ AIC, change in Akaike information criterion relative to a demographics only model (age and sex). In CU, tau PET refers to [^{18}F]RO948 SUVR at baseline in the EBM stage I ROI; in MCI, tau PET refers to [^{18}F]RO948 SUVR at baseline in the EBM stage II ROI.

© 2021 Leuzy A et al. *JAMA Neurology*.

eTable 20. Relationship between individual biomarkers and tau PET using EBM ROIs and CSF biomarkers in Aβ-positive CU and Aβ-positive MCI from BioFINDER-1

Group	Predictor	Coefficient	Adjusted R^2	P	Δ AIC
Aβ+ CU	CSF Aβ42/40	0.02	0.00 [-0.02, 0.02]	0.9017	2
	Amyloid PET	0.36	0.13 [-0.08, 0.33]	0.0416	-2
	CSF p-tau181	0.54	0.27 [0.16, 0.42]	0.0039	-13
	Tau PET	0.38	0.14 [-0.07, 0.36]	0.0378	-2
	CSF NFL	0.16	0.02 [-0.08, 0.13]	0.4240	1
	Hippocampal volume	-0.10	0.01 [-0.05, 0.07]	0.6321	2
	AD cortex	0.03	0.00 [-0.02, 0.02]	0.8751	2
Aβ+ MCI	CSF Aβ42/40	0.29	0.08 [-0.12, 0.27]	0.2466	0
	Amyloid PET	0.20	0.04 [-0.10, 0.18]	0.4260	1
	CSF p-tau181	0.48	0.22 [-0.06 0.49]	0.0346	-3
	Tau PET	0.59	0.33 [0.04, 0.62]	0.0094	-6
	CSF NFL	0.05	0.00 [-0.04, 0.04]	0.8313	2
	Hippocampal volume	-0.16	0.02 [-0.09, 0.14]	0.5299	2
	AD cortex	-0.13	0.02 [-0.08, 0.11]	0.6142	2

Aβ+, amyloid-β positive; CU, cognitively unimpaired; MCI, mild cognitive impairment; Aβ42/40, ratio of Aβ42 to Aβ40; PET, positron emission tomography; p-tau181, tau phosphorylated at threonine 181; NfL, neurofilament light; AD cortex, cortical thickness in a temporal meta-ROI encompassing temporal regions with known susceptibility in AD; R^2, coefficient of determination; P, p-value; Δ AIC, change in Akaike information criterion relative to a demographics only model (age and sex). In Aβ+ CU, tau PET refers to [^{18}F]flortaucipir SUVR at baseline in the EBM stage I ROI; in Aβ+ MCI, tau PET refers to [^{18}F]flortaucipir SUVR at baseline in the EBM stage II ROI. Amyloid PET refers to the SUVR at baseline in a neocortical meta-ROI, expressed in Centiloids.

© 2021 Leuzy A et al. *JAMA Neurology*.

eReferences

1. American Psychiatric Association., American Psychiatric Association. Task Force on DSM-IV. *Diagnostic and statistical manual of mental disorders : DSM-IV.* 4th ed. Washington, DC: American Psychiatric Association; 1994.
2. Jack CR, Jr., Bennett DA, Blennow K, et al. NIA-AA Research Framework: Toward a biological definition of Alzheimer's disease. *Alzheimers Dement.* 2018;14(4):535-562.
3. Haftenberger M, Schuit AJ, Tormo MJ, et al. Physical activity of subjects aged 50-64 years involved in the European Prospective Investigation into Cancer and Nutrition (EPIC). *Public Health Nutr.* 2002;5(6B):1163-1176.
4. McKhann GM, Knopman DS, Chertkow H, et al. The diagnosis of dementia due to Alzheimer's disease: recommendations from the National Institute on Aging-Alzheimer's Association workgroups on diagnostic guidelines for Alzheimer's disease. *Alzheimers Dement.* 2011;7(3):263-269.

© 2021 Leuzy A et al. *JAMA Neurology*.
